# The interaction between LC8 and LCA5 reveals a novel oligomerization function of LC8 in the ciliary-centrosome system

**DOI:** 10.1038/s41598-022-19454-4

**Published:** 2022-09-16

**Authors:** Tamás Szaniszló, Máté Fülöp, Mátyás Pajkos, Gábor Erdős, Réka Ágnes Kovács, Henrietta Vadászi, József Kardos, Zsuzsanna Dosztányi

**Affiliations:** 1 Department of Biochemistry, Institute of Biology, ELTE Eötvös Loránd University, Budapest, Hungary; 2 ELTE NAP Neuroimmunology Research Group, Department of Biochemistry, Institute of Biology, ELTE Eötvös Loránd University, Budapest, Hungary

**Keywords:** Intrinsically disordered proteins, Biochemistry, Cytoskeletal proteins

## Abstract

Dynein light chain LC8 is a small dimeric hub protein that recognizes its partners through short linear motifs and is commonly assumed to drive their dimerization. It has more than 100 known binding partners involved in a wide range of cellular processes. Recent large-scale interaction studies suggested that LC8 could also play a role in the ciliary/centrosome system. However, the cellular function of LC8 in this system remains elusive. In this work, we characterized the interaction of LC8 with the centrosomal protein lebercilin (LCA5), which is associated with a specific form of ciliopathy. We showed that LCA5 binds LC8 through two linear motifs. In contrast to the commonly accepted model, LCA5 forms dimers through extensive coiled coil formation in a LC8-independent manner. However, LC8 enhances the oligomerization ability of LCA5 that requires a finely balanced interplay of coiled coil segments and both binding motifs. Based on our results, we propose that LC8 acts as an oligomerization engine that is responsible for the higher order oligomer formation of LCA5. As LCA5 shares several common features with other centrosomal proteins, the presented LC8 driven oligomerization could be widespread among centrosomal proteins, highlighting an important novel cellular function of LC8.

## Introduction

Hub proteins play a central role in coordinating a vast array of cellular processes by being able to interact with a large number of proteins^1–3^. In many cases, hub proteins recognize specific sequence elements called short linear motifs (SLiMs) that typically reside within intrinsically disordered regions of partner proteins^4,5^. Dynein light chain LC8 is an 89-residue-long, highly conserved eukaryotic hub protein^6^. It forms a homodimer presenting two parallel and identical binding grooves on opposite sides of the dimer that interact with 8-residue-long SLiMs^4,7,8^. The known motif instances are versatile but typically contain a TQT core sequence, or at least two out of these three residues^9^. Till today, over 100 binary interactions of LC8 have been described, and bioinformatics motif filter protocols suggest that there could be many more LC8 partners in the human proteome^10,11^. Known binding partners are involved in a wide range of functions that include intracellular and nuclear transport, apoptosis, autophagy and cell division^9,12^. Despite the extensive interaction network, many aspects of LC8 function are still unresolved.

LC8 was first described as a light chain of the dynein motor complex^13^, binding the two intermediate chains of the complex^14,15^. Based on this association, LC8 was originally suggested to function as a cargo adaptor of the motor complex^16^. However, simultaneous binding of the intermediate chain and other partners that would support this model, has not been observed. Currently, the most accepted theory explaining the function of LC8, is that it is a hub driving the dimerization of partner proteins. Several of its known partners contain coiled coil regions, which are formed only as a result of LC8 binding^17,18^. LC8 was also suggested to promote the formation of tetramers or even higher order oligomers. This was observed in the case of Anastral spindle 2 (ANA2), a Drosophila specific centrosomal protein, and the human NIMA related protein kinase 9 (NEK9). Slevin and coworkers found that LC8 is necessary for the dimerization of ANA2 stretches, and the ANA2 dimers are further tetramerized through coiled coil interaction^18^. The interaction of LC8 with Nek9 also increased the propensity of coiled coil interaction-driven oligomerization and regulated the activity of the enzyme. However, LC8 binding was not required for dimerization^19^.

In the last few years, the expanding interaction network of LC8 highlighted the possibility of novel functions and drew attention to the potential role of LC8 in the ciliary-centrosomal system. Centrosomes and the basal body of the primary cilium are the major microtubule-organizing centers of animal cells. The primary cilium and the centrosomal system incorporate numerous large, coiled coil forming proteins predicted to contain long disordered regions^20–22^. These proteins are responsible for maintaining the structural integrity of the ciliary-centrosomal system. Their interaction network also provides a platform for other components to interact with each other and orchestrate the processes responsible for cell signaling events, cytoskeletal organization, cell polarity, and cell division^23,24^. Interestingly, several comprehensive high-throughput interaction data connect LC8 to the ciliary-centrosomal system^25–29^. The SLiMs responsible for the direct binding of LC8 have been identified only in a few cases^11,18,30^. Overall, our knowledge about the function of LC8 is limited in this system.

Our motif filtering protocol suggested a putative LC8 binding motif in a 697 amino acid long protein lebercilin (LCA5, also known as Leber congenital amaurosis 5^10^. LCA5 is localized in the ciliary basal body, ciliary axoneme of the primary cilia and the connecting cilia of photoreceptor cells^31^. It is a known ciliopathy gene whose mostly truncating mutations can cause Leber Congenital Amaurosis, a severe degenerative disease of the photoreceptor cells which leads to blindness at an early age^31,32^. Boldt and co-workers found an interaction between LCA5 and LC8 using a SILAC/AP-based approach and suggested that the region responsible for the LC8 binding is localized on the C-terminal part of the protein after residue 279^33^. Nevertheless, the LCA5-LC8 interaction was not characterized in detail and the binding motifs have not been identified. In the present work, we validate and characterize the linear motif-based interaction between LC8 and LCA5. As studies on linear motif interactions are highly prone to provide both false positive and false negative results^34^, we use a multifaceted approach to verify the interaction at the cellular, protein and motif level. Thus, we provide a detailed biophysical characterization of the interaction and propose a novel function and binding model for LC8-mediated interactions, which could be relevant for similar proteins, especially in the centrosomal system.

## Results

### LCA5 and LC8 interact under cellular conditions and colocalize in the ciliary basal body and in the centrosome

**Figure 1:**
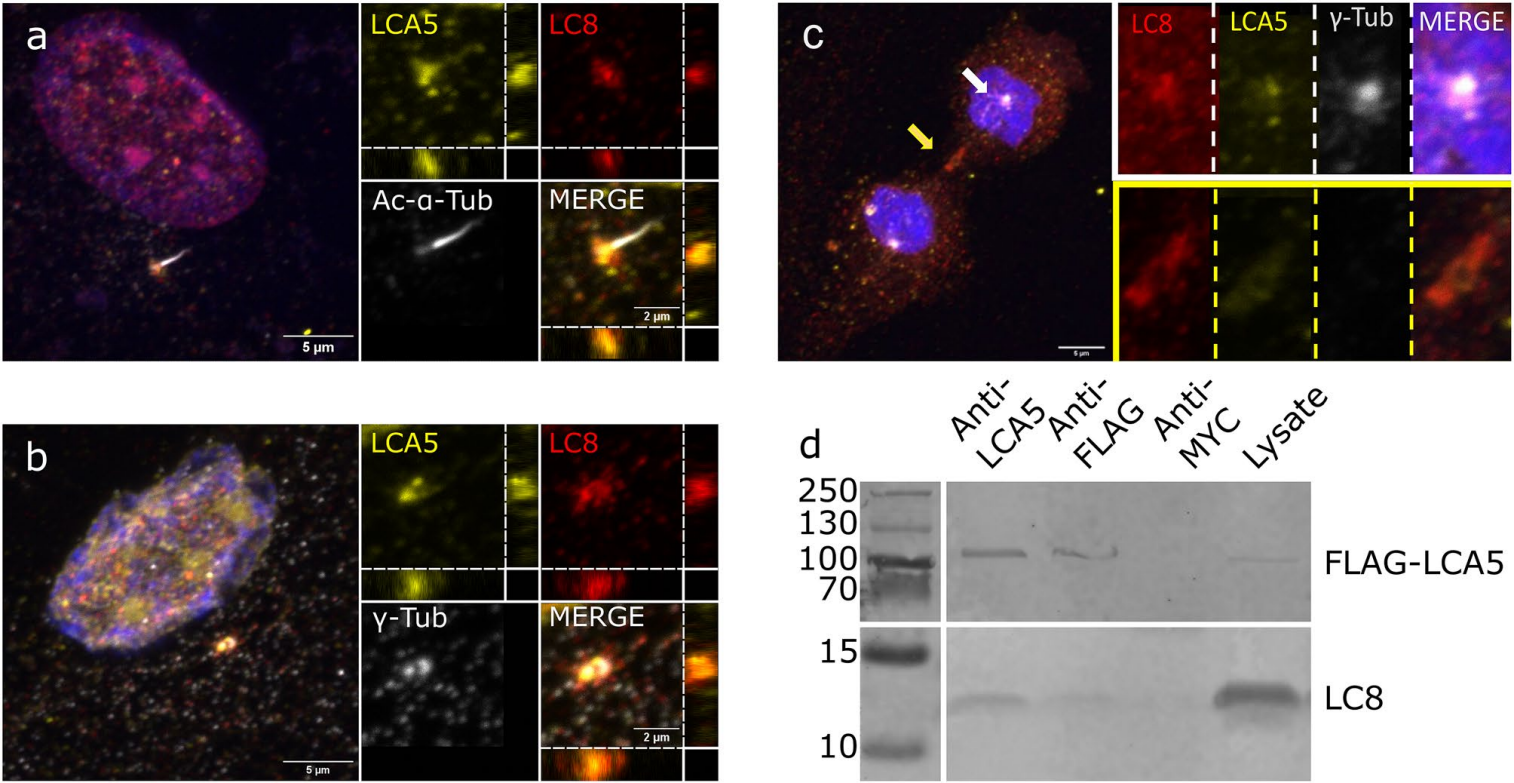
Colocalization and co-immunoprecipitation of LC8 and LCA5. (**a**) LCA5 and LC8 colocalize at the ciliary basal body of hTERT-RPE1 cells, with zx and zy cross-sections at the basal body. The brightness of the intersections was adjusted for better visibility (**b**) Colocalization in the centrosome in late interphase cells at both the mother and daughter centrioles. A zx and zy cross-section shows the overlapping at the centrioles. The brightness of the intersections was adjusted for better visibility **(c**) In dividing cells, LCA5 and LC8 show a strong colocalization around the centrioles (white framed insert) and in the midbody (yellow framed insert). (**d**) Co-immunoprecipitation of LC8 with full-length FLAG-LCA5 in HEK293 cells. The FLAG-LCA5-LC8 interaction was detected by immunoprecipitation with anti-LCA5 and anti-FLAG antibodies, but no interaction was observed using the control antibody anti-MYC. The original image was cropped for better visibility (see the original blot in Supplementary Fig. S3).

We performed colocalization experiments to identify the cellular localization of LCA5-LC8 complexes. In accordance with previous data^31^, our results showed a strong LCA5 localization at the basal body of primary cilia (Fig. 1a) and around the centrioles of the centrosomes in late interphase hTERT-RPE1 immortalized retinal pigment epithelial cells (Fig. 1b). Furthermore, our results also showed that within the primary cilia, the localization of LCA5 was more abundant at the ciliary tip than in the whole axoneme (Fig. 1a). LC8 showed a strong localization to the basal body of ciliated cells and around the centrioles in the centrosome of non-ciliated cells. (Fig. 1a, b). LCA5 and LC8 showed colocalization at the centrosome (Pearson’s correlation coefficient: 0.802 thresholded Manders for LCA5 to LC8 tM1: 0.929 and LC8 to LCA5 tM2: 0.574) in late interphase cells and at the ciliary basal body in ciliated cells (Pearson’s correlation coefficient: 0.715 thresholded Manders for LCA5 to LC8 tM1: 0.671 and LC8 to LCA5 tM2: 0.718) (Fig. 1a, 1b, Supplementary Fig. S1, S2). The absence of colocalization in the ciliary axoneme indicates that the interaction is not related to the axonemal transport machinery, but specifically connected to the centrosome-basal body system. Interestingly, LCA5 and LC8 also showed overlap in the mitotic spindle and in the midbody during cytokinesis (Fig. 1c).

In order to confirm the interaction at full-length protein level in a cellular environment between LCA5 and LC8, a co-immunoprecipitation experiment was carried out. For this, FLAG-tagged full-length LCA5 and HA-tagged LC8 were co-expressed in HEK-293 (human embryonic kidney) cell line. LC8 was co-precipitated with FLAG-tagged LCA5 bait using both anti-LCA5 and anti-FLAG antibodies, but no interaction was observed in the immunoprecipitate of control antibody (anti-MYC) (Fig. 1d, see the original western blot in Supplementary Fig. S3). Altogether, these results confirm that LCA5 and LC8 colocalize in the ciliary basal body and in the centrosome, and could directly interact in a cellular environment.

### LC8 interacts with LCA5 via short linear motifs

**Figure 2:**
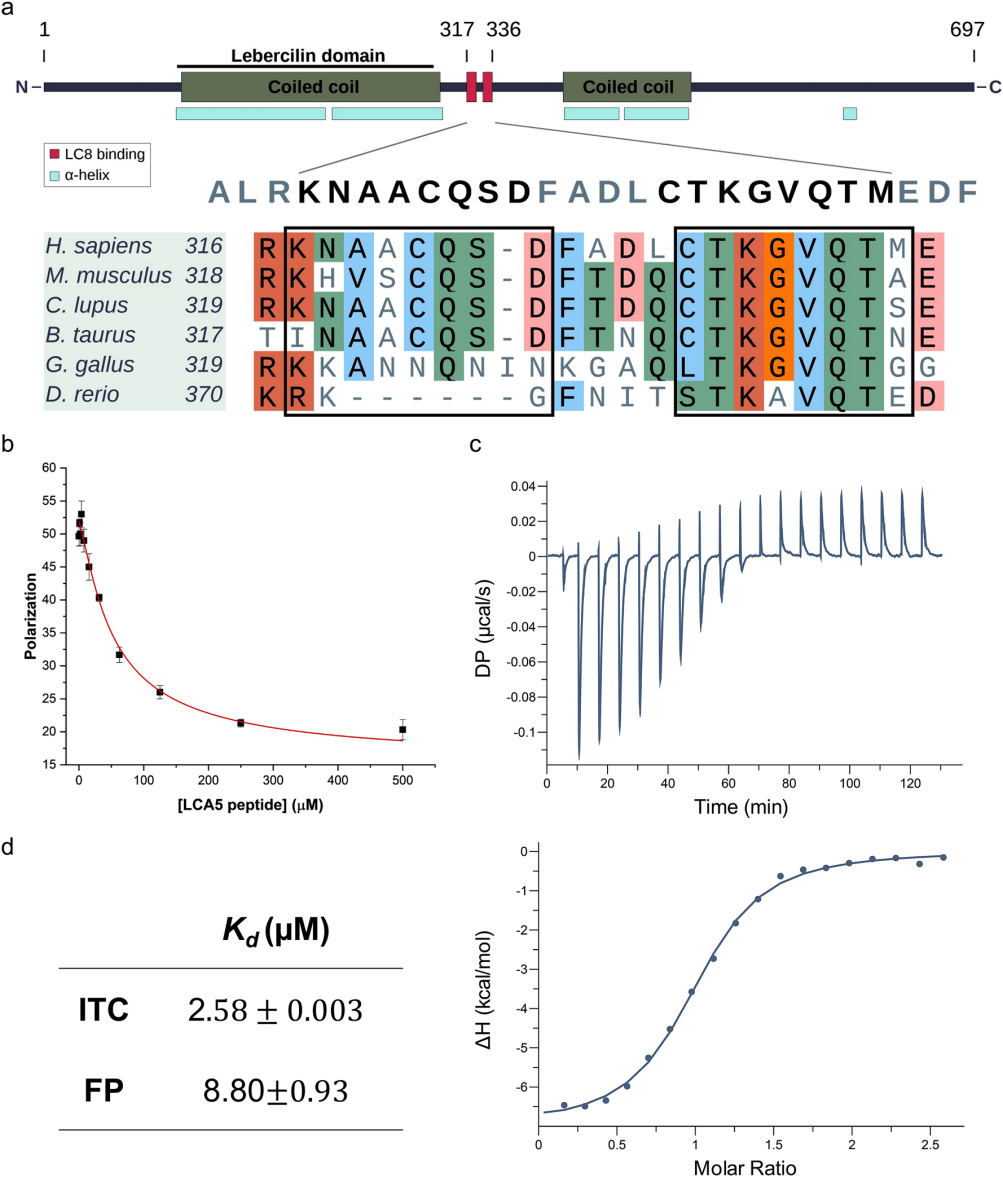
Sequence localization of the binding motifs in LCA5 and the interaction of the VQT motif with LC8. (**a**) The domain structure of the full-length LCA5 and multiple sequence alignment of the two putative LC8 binding motifs generated from the corresponding LCA5 orthologs. The high confidence score VQT motif is conserved through vertebrates, the lower score CQS core triplet-containing motif only emerged among mammals. (**b**) In competitive fluorescence polarization measurement, LCA5 binding motif-containing synthetic peptide (DLCTKGVQTME) was added to the complex of LC8 with fluorescein labeled BMF peptide. (**c**) ITC thermogram of LCA5 peptide injected to LC8 protein. (**d**) Comparison of the resulting K_d_-s of the LCA5 peptide—LC8 interaction from the two methods.

Next, we sought to identify the specific binding region of LCA5. In general, LC8 recognizes short linear motifs located within intrinsically disordered regions. The structural features of LCA5 have not yet been experimentally characterized. However, bioinformatic prediction indicates that it contains no globular domains but only long disordered segments and coiled coil regions^31^ (Supplementary Fig. S4). The most conserved part of the sequence, which corresponds to the Lebercilin PFAM family, overlaps with the N-terminal coiled coil region. The C-terminal part of the sequence is predicted to be largely disordered with additional coiled coil-forming parts (Fig. 2a).

Our motif filtering protocol ^10^—developed to highlight high-confidence interaction motifs for LC8—identified a putative binding motif corresponding to residues 329–336 (CTKGVQTM) (Fig. 2a). This motif is located within a predicted disordered region (Supplementary Fig. S4) and shows high conservation at the level of vertebrates (Fig. 2a), further supporting the biological relevance of the motif. The 11-residue-long synthetic peptide (DLCTKGVQTME) of LCA5 containing the putative binding motif was tested in a competitive fluorescence polarization binding assay against the Bcl-2-modifying factor (BMF) motif, a well-characterized partner of LC8^35^ (Fig. 2b, d). The FP assay resulted in an 8.1 µM dissociation constant (K_d_) which is in the typical range of SLiM-based interactions of LC8. We performed isothermal titration calorimetry (ITC) as an alternative method that provided a very similar result (K_d_ = 2.6 μM) (Fig. 2c, d).

At a closer inspection of the sequence, we found an additional low-scoring motif N-terminally of the characterized VQT motif, which corresponds to residues 317–324 of LCA5 (KNAACQSD) (Fig. 2a, b). The two motifs are located at 4 residue distance to each other. However, according to the model based on the intraflagellar dynein 2 motor complex^30^, this spacing is sufficient for the simultaneous binding of two LC8 molecules (Supplementary Fig. S4). However, the binding of the second motif to LC8 could not be verified at the peptide level.

### GST pull-down reveals the ability of a second binding motif to bind LC8 at the protein level

**Figure 3:**
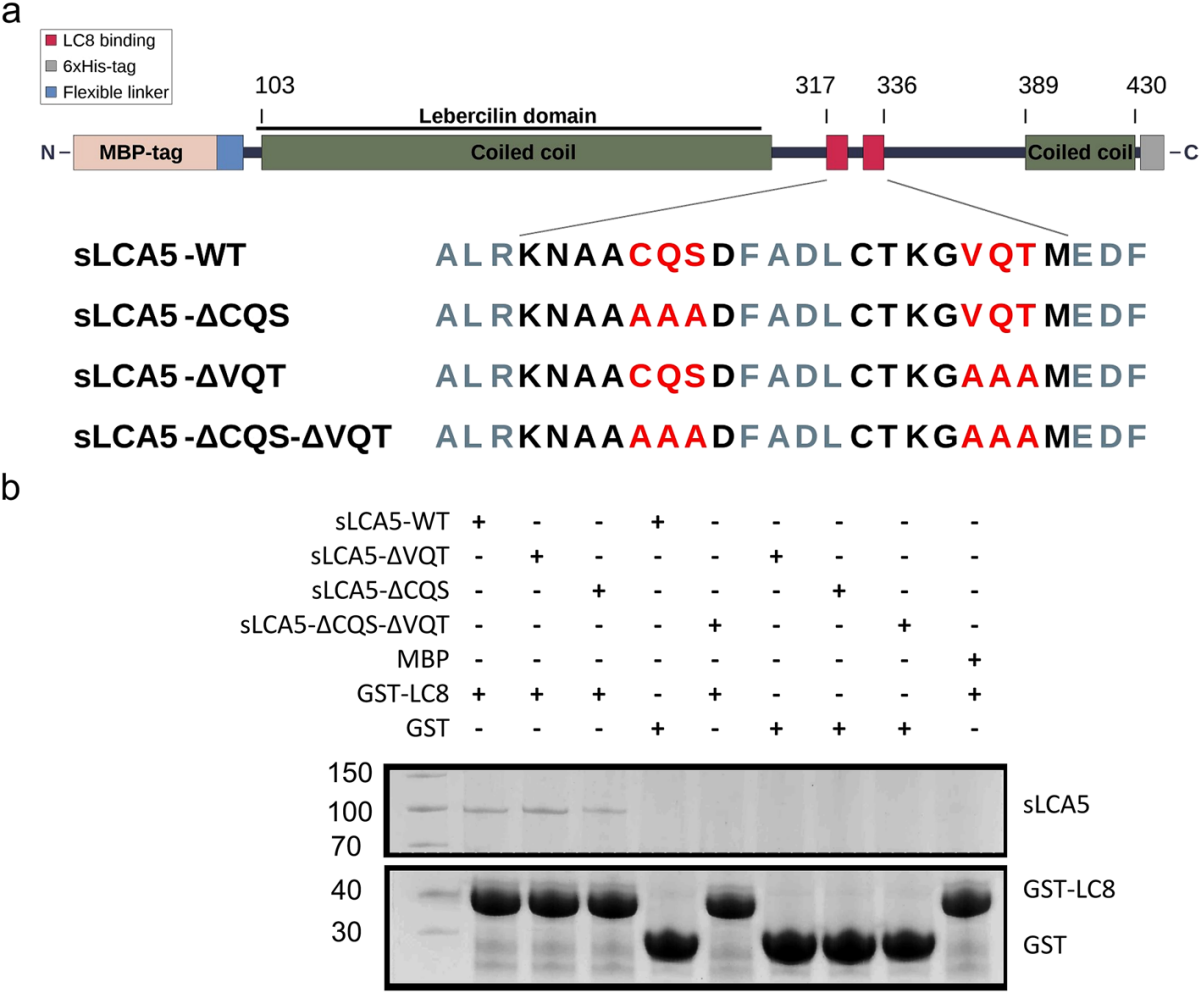
The scheme of the LCA5 recombinant protein constructs and GST pull-down assay. (**a**) Wild-type and mutant constructs with alanine mutations in the core triplets of the LC8 binding regions. An N-terminal MBP tag, connected with a flexible linker region, was introduced to maintain the solubility of the construct. (**b**) In vitro GST pull-down of LC8 and sLCA5 with the mutation analysis of LCA5 binding motifs. The wild type and both of the single binding motif-mutant sLCA5 forms can bind LC8, whereas the introduction of mutations in both putative binding motifs was able to hinder the interaction (see the original gel in Supplementary Fig. S5).

In order to prove the necessity of the motif for the interaction above the peptide motif level, we carried out a GST pull-down experiment using wild type and mutated LCA5 protein fragments. We used a truncated construct of LCA5 (LCA5^96–430^ referred further as sLCA5-WT) fused to an N-terminal MBP tag to achieve better solubility and stability in our in vitro experiments (Fig. 3a).

In the mutant sLCA5-ΔVQT, the three core residues (VQT) of the predicted binding motif CTKGVQTM were exchanged for three alanines (Fig. 3a). Unexpectedly, the mutation did not abolish the interaction with LC8 (Fig. 3b and see original gel in Supplementary Fig. S5). To exclude the possibility that the exchange of central motif residues VQT to alanines was not enough to impair the interaction, we introduced further alanine mutations into the motif (CAAAAAAM). The subsequent GST pull-down assay showed that interaction between LCA5 and LC8 persisted despite the mutations (Supplementary Fig. S6). We also tested binding through the other CQS motif by additional pull-down experiments. Therefore, we created two additional mutant constructs, the sLCA5-ΔCQS, and the double mutant sLCA5-ΔCQS-ΔVQT (Fig. 3a). The results of these pull-down experiments showed that both of the single mutants maintained the interaction with LC8 and only mutations introduced into both motifs abolished the interaction with LC8 (Fig. 3b). These results indicate that although only one of the motifs could be validated at the peptide level, both motifs are capable of binding LC8 in the context of the protein.

### ITC experiments reveal unusual binding stoichiometries

**Figure 4:**
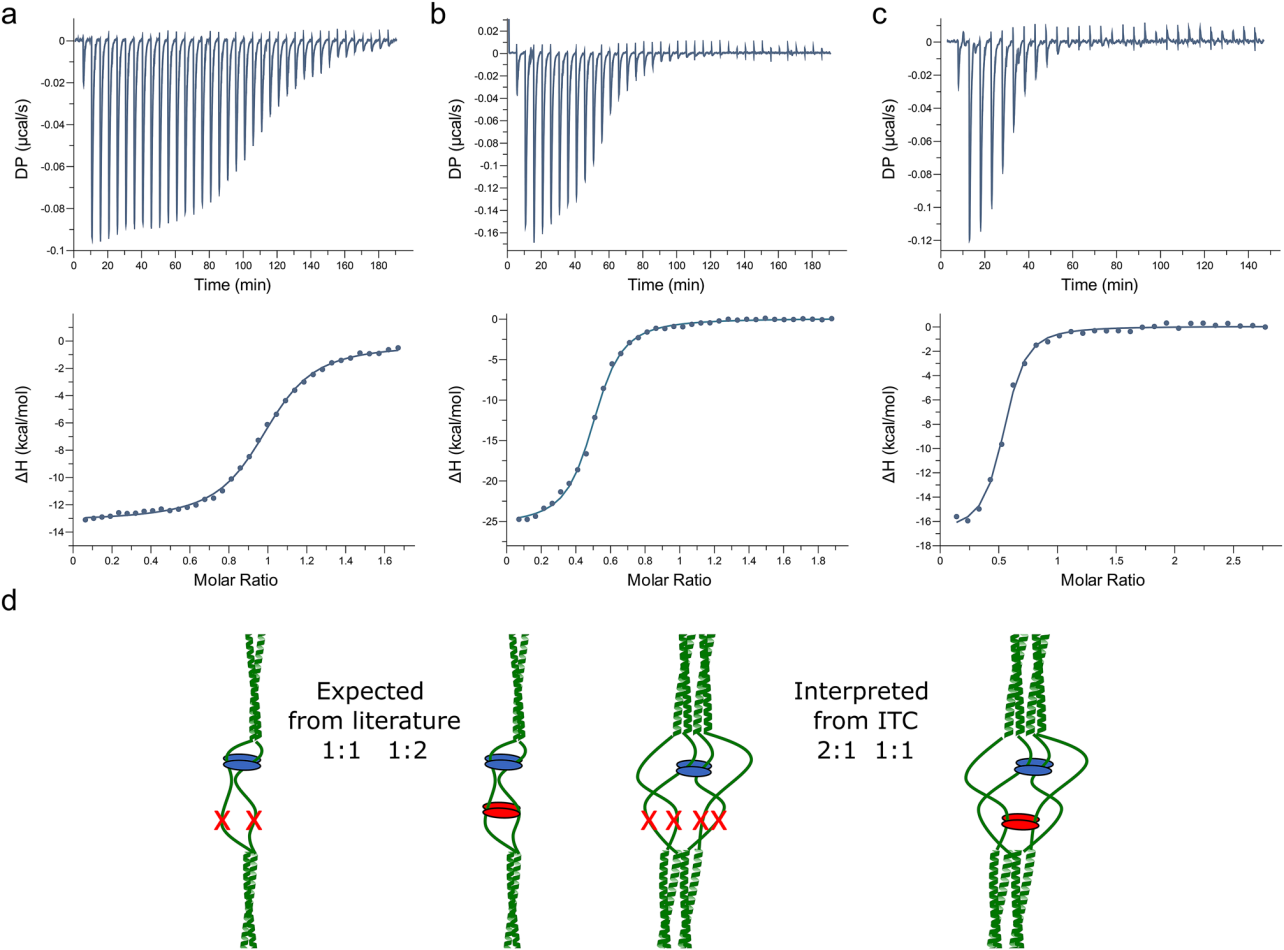
ITC measurements of sLCA5-LC8 complexes. Representative thermograms of (**a**) sLCA5-WT (**b**) sLCA5-ΔCQS and (**c**) sLCA5-ΔVQT (**d**) Comparison of the possible compositions of LC8 complexes according to the accepted dimerization model and the non-saturated tetrameric complexes indicated by our ITC measurements. In case of dimeric complexes with one LC8 binding site the expected monomeric ratio is 1:1 and with two LC8 binding sites the ratio should be 1:2 (see also Supplementary Table S1). However, in the case of LCA5, the single mutants of LC8 containing only one binding motif and the wild type containing two binding sites show an unexpected 2:1 and 1:1 ratio, respectively, according to our ITC measurements. In the case of single mutant LCA5, the calculated minimal stoichiometry indicates a tetrameric LCA5 complex with only one of the LC8 binding site pairs occupied. Based on this, it is very likely that the wild type LCA5 could incorporate LC8 dimers at both binding sites.

Next we carried out a series of ITC experiments to determine the affinities and the stoichiometries of LCA5-LC8 interaction. We tested the wild-type and the three mutant forms of the fragment sLCA5 (Fig. 3a). The wild-type sLCA5-WT fragment bound LC8 with an overall K_d_ of 0,76 μM (Fig. 4a, Table 1). Among the two single mutant forms, the VQT motif (sLCA5-ΔCQS) had the stronger binding with a K_d_ value of 0.68 μM, while the binding constant for the sLCA5-ΔVQT fragment was 1.22 μM (Fig. 4b, c, Table 1). In all three interactions, the binding was enthalpically favorable but entropically unfavorable. The entropic contribution was the most notable in the case of the interaction via the VQT containing motif (Table 1). The double mutant sLCA5-ΔCQS-ΔVQT titrated with LC8 did not show any measurable interaction.

**Table 1:** Measured binding affinities, thermodynamic properties, and binding stoichiometries. Thermodynamic and kinetic parameters of interaction between LC8 and wild type and single mutant forms of sLCA5 and their corresponding stoichiometries.

	K_d_ (µM)	ΔH (kcal/mol)	ΔG (kcal/mol)	− TΔS (kcal/mol)	Stoichiometry LCA5:LC8
sLCA5-WT	0.76 ± 0.06	− 11.80 ± 0.19	− 8.49	3.28	1:1
sLCA5-ΔCQS	0.68 ± 0.08	− 8.62 ± 0.14	− 8.55	0.07	2:1
sLCA5-ΔVQT	1.22 ± 0.08	− 12.50 ± 0.11	− 8.20	4.32	2:1

According to the standard model of LC8 interactions, the dimeric interface of LC8 presents two interaction sites for partners which also form dimers, implying a 1:1 stoichiometry. Partners with two adjacent binding motifs, like ANA2, follow a 1:2 stoichiometry by binding two LC8^18^. Intriguingly, we observed a 2:1 LCA5:LC8 stoichiometry for the ΔCQS and the ΔVQT mutants, which means that one LC8 dimer binds four LCA5 chains (Supplementary Table S1). A reasonable interpretation is that the native LCA5 is dimeric and thus a pair of LCA5 dimers bind to one dimeric LC8. In such a situation, one interacting site remains free on one chain of each LCA5 dimer. It is possible that after LC8 binding on one chain of an LCA5 dimer, the binding site is sterically hindered on the other chain. In the case of the wild-type sLCA5-WT, a 1:1 LCA5:LC8 stoichiometry was observed (Fig. 4b, c, Table 1). Because the number of binding sites in sLCA5-WT is doubled compared to the single binding site mutants, it is plausible that this can bind twice the amount of LC8 molecules, resulting in 1:1 stoichiometry. Figure 4d shows the different scenarios based on the expected and observed stoichiometries To support this hypothesis, further experiments are needed to verify that LCA5 molecules could form dimers in the absence of LC8.

### LCA5 forms coiled coil oligomers in an LC8 independent manner, and LC8 stabilizes its structure

**Figure 5:**
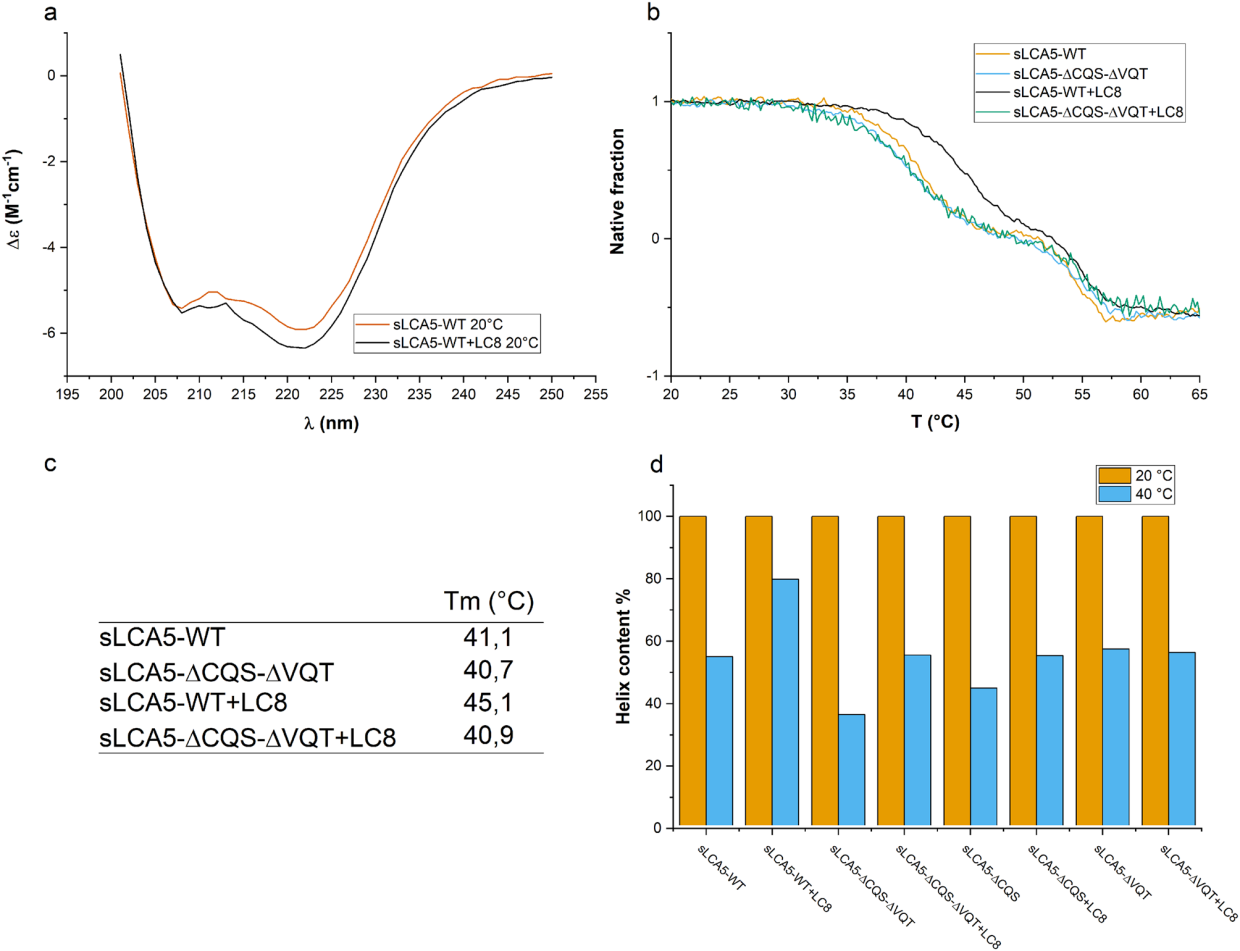
Structural characterization of sLCA5-LC8 complexes by CD spectroscopy (**a**) The CD spectra of sLCA5-WT and sLCA5-WT in complex with LC8 at 20 °C. The measured spectra indicate a strong coiled coil forming propensity in both cases with very similar helical content. (**b**) The temperature-dependent unfolding of sLCA5-WT, sLCA5-ΔCQS-ΔVQT, and LC8 in complex with sLCA5 -WT and sLCA5-ΔCQS-ΔVQT. The presented normalized graph includes only the two first phases of unfolding, namely the 1–0 region on the Y-axis is the unfolding of LCA5, below 0 represents the unfolding of the MBP fusion tag, LC8 was melted above 70 °C (not presented). (**c**) Melting temperatures of the wild-type and double mutant form of sLCA5 without and in complex with LC8. The melting temperatures were similar in case of the double mutants and the wild type form without LC8. The melting temperature of sLCA5^-^WT in complex with LC8 was greatly increased around physiological temperatures. (**d**) The calculated helix content (%) of each LCA5 construct with and without LC8 at 20 °C and 40 °C.

To analyze the secondary structure composition of LCA5 alone and the structural changes induced upon LC8 binding, we carried out CD spectroscopy measurements. The CD spectra of the wild-type LCA5 showed the formation of long α-helices indicated by the local minimum at 208 nm, and the more pronounced absolute minimum at 222 nm, which is characteristic of coiled coil formation (Fig. 5a). The CD measurements of the sLCA5-ΔCQS-ΔVQT and the single mutant constructs also led to similar α-helical properties with a strong indication of coiled coil formation without any traces of decreased structural integrity caused by the introduced mutations (Supplementary Fig. S8). The CD spectra with pronounced α-helical content and the strong propensity for extensive coiled coil formation confirms the per se dimerization of LCA5.

By the addition of LC8 to sLCA5-WT construct in fivefold molar excess, the measured molar ellipticity curve remained the same at 20 °C as sLCA5-WT without LC8, indicating that binding of LC8 did not induce further coiled coil formation (Fig. 5a). At higher temperatures (at 37 °C and 40 °C), the α-helical content decreased. However, sLCA5-WT in complex with LC8 maintained its original helical content far better than the same construct without LC8 (Supplementary Fig. S9). The addition of LC8 showed no effect on the dimerization of LCA5, but effectively maintained the structural integrity of the complex.

We also carried out a thermal denaturation measurement series in the range of 10–80 °C on sLCA5-WT, sLCA5-ΔCQS-ΔVQT, and these in complex with LC8 to study its contribution to structural stability. Without LC8, the wild-type and double mutant LCA5 constructs showed very similar thermal denaturation profiles with a two-stage melting curve (Fig. 5b). The wild-type LCA5 in complex with LC8 showed a ~ 5 °C increase in the melting temperature (Fig. 5b, c). This structure stabilizing effect was the direct consequence of the interaction between LC8 and LCA5 as no similar increase was observed in case of double mutant LCA5 in the presence of LC8. Without LC8 binding, the structure destabilization began at 35 °C and rapidly increased around the normal physiological temperature range. The binding of LC8 shifted the beginning of this structural decay above the normal physiological temperature. In accordance with the thermal denaturation profiles, alpha-helical content calculated from the CD spectra at 20 and 40 °C lead to similar observations (Fig. 5d). Between 20 and 40 °C the LCA5 constructs without LC8 lost at least 50% of their helical residue content. Similar behavior was observed in the presence of LC8 for all constructs except the sLCA5-WT which preserved 80% of its helical structures at higher temperatures (Fig. 5d).

### LC8 binding enhance the oligomerization of LCA5

Next, we analyzed the molecular size of the fractions of LCA5 in complex with LC8 by analytical gel filtration to determine how LC8 enhances the oligomerization capabilities of the various constructs. The elution profile of the complex of the wild type LCA5 fragment with LC8 showed three pronounced peaks.

**Figure 6:**
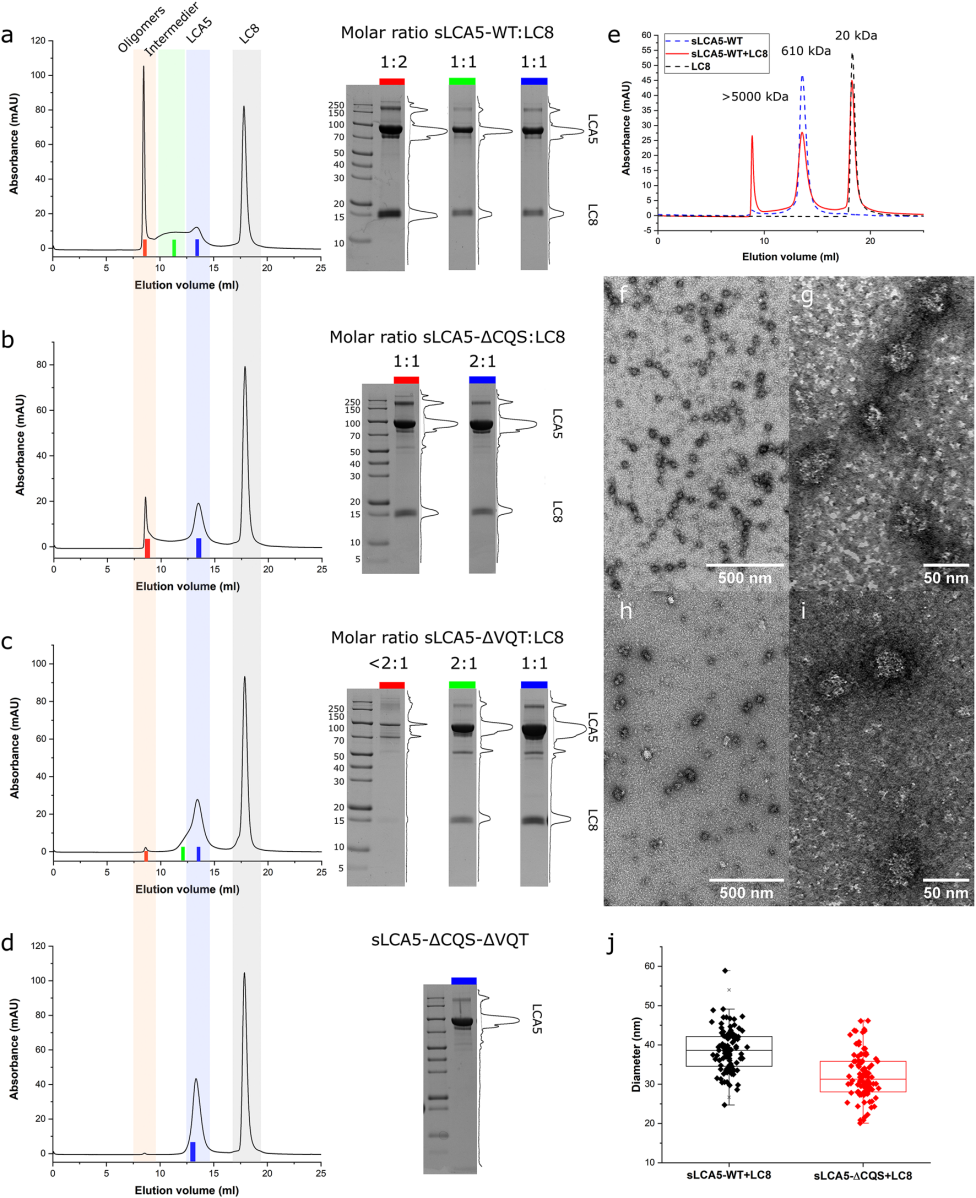
Characterization of LCA5 oligomers. (**a**–**d**) The chromatograms of the sLCA5 variants in complex with LC8 and SDS-PAGE analysis of the selected fractions. The light colored sections of the chromatograms represent the major peak types, the oligomeric fraction (light red) in case of the sLCA5-WT, the intermedier fraction (light green), the LCA5 fraction (light blue) and the LC8 fraction (light gray). The colored markers represent the collected fractions and correspond to the SDS-PAGE gels used for densitometry. The oligomeric fraction of the sLCA5-WT and the sLCA5-ΔCQS (red marker) contain LC8 at the molar ratio of 1:2 and 1:1. The intermediate fraction of sLCA5-WT (green) contains LC8 at the molar ratio of 1:1. The LCA5 fractions of sLCA5-WT (blue), sLCA5-ΔCQS (blue), sLCA5-ΔVQT (blue) contain LC8 at the molar ratio of 1:1, and 2:1 respectively. In the case of sLCA5-ΔVQT, the blue marked sample contains saturated sLCA5 complexes at the molar ratio of 1:1. The sLCA5-ΔCQS-ΔVQT was unable to bind LC8 and contains only the double mutant sLCA5 (see the original gels in Supplementary Fig. S13-15). (**e**) Merged chromatograms of sLCA5-WT (blue dashed line 610 kDa), LC8 (black dashed line 20 kDa) and sLCA5-WT in complex with LC8 (red line), which contains the oligomeric fraction (> 5000 kDa), the LCA5 and LC8 peaks. The short incubation time results in decreased oligomeric fraction and the lack of intermediate forms detected between the LCA5 peak and the oligomeric peak. (**f**–**i**) Representative TEM images of sLCA5-WT and sLCA5-ΔCQS in complex with LC8, at 50,000, and 300,000-fold magnification. In both cases, spherical oligomeric complexes were detected with diameters of 30–40 nm. The size distribution and the overall number of the oligomers differs between the two complexes (**j**). The diameters of the sLCA5-WT and sLCA5-ΔCQS oligomers are compared by two sample t-test and significantly differ from each other at *p* = 0.05 significance level. The diameters of the two oligomer populations showed normal distribution by Saphiro-Wilk test at *p* = 0.05.

The smallest molecular weight peak corresponded to LC8, with an estimated molar mass of ~ 20 kDa, in good agreement with the molar mass calculated from the sequence assuming a dimeric LC8 (20.7 kDa) (Fig. 6e). The next fraction in molecular weight corresponded to LCA5 showing an estimated molar mass of ~ 610 kDa. This size corresponds to octamers of the 84 kDa monomeric LCA5 constructs, but is likely to be an overestimation due to the elongated shape of the coiled coil forming LCA5 molecules. The elution volume of the native and complexed wild-type LCA5 construct showed no significant difference, similarly to the single mutant constructs (Fig. 6e, Supplementary Fig. S10). This suggests that the most likely low oligomeric state of LCA5 is tetrameric, which is in agreement with the minimal stoichiometric form measured by ITC (Table 1).

Interestingly, there was a high molecular weight fraction observable that eluted in the over 5000 kDa void volume, indicating the presence of higher order oligomers (Fig. 6a–e, Supplementary Fig. S10, S11). The size of this peak was dependent on the LCA5 variant and if LC8 was added. The void fraction was negligible without LC8 and also when both motifs or the stronger VQT motif was mutated. The addition of LC8 to the sLCA5-WT construct in fivefold molar excess led to a remarkable increase in the void volume fraction which was also detected in the case of the sLCA5-ΔCQS LC8 complex, but to a smaller extent. Comparing the size of the void volume fractions, the wild type sLCA5 had an almost ~ 20% stronger propensity to form higher order oligomers than the sLCA5-ΔCQS (Fig. 6a–b, Supplementary Fig. S12).

In order to gain further insights into the oligomerization process, we repeated the experiment with increased incubation time. The prolonged incubation of the complexes remarkably increased the void fraction size, especially for the wild-type complex where the increment was 324%, while the increment was only 35% for the sLCA5-ΔCQS construct compared to the non-incubated complexes. The difference between the incubated complexes of the wild type sLCA5 and the sLCA5-ΔCQS increased ~ 3.5 fold. (Supplementary Fig. S12). We performed SDS-PAGE and densitometric analysis of the fractions at selected stages of the gel filtration experiments, which enabled us to characterize the sLCA5-LC8 molar ratios. This confirmed that LC8 was present in the oligomeric forms. The LC8 binding sites of sLCA5-WT and the sLCA5-ΔCQS complexes were saturated in the oligomeric fractions, with a molar ratio of the sLCA5 and LC8 was 1:2 and 1:1, respectively. In the case of the sLCA5-ΔVQT, we detected only a trace amount of LC8 and the complex should be far from saturation (Fig. 6a–c).

The increased incubation time of the complexes lead to the appearance of additional states which can present different stoichiometric compositions. The wild type construct showed an elongated peak shape on the elution volume. This is likely to correspond to a mixed fraction of multiunit oligomers between the size of the likely tetrameric LCA5 fraction and the higher oligomeric fraction. Despite its oligomeric state, the LCA5-LC8 molar ratio was 1:1, indicating that the LC8 binding sites were not saturated in accordance with the ITC stoichiometries (Fig. 6a). The LCA5 peak of the sLCA5-WT and the sLCA5-ΔCQS complexes had the same elution volume as the single constructs, however these fractions contained LC8 in the molar ratio of 1:1 and 2:1, respectively (Fig. 6a–b). The LCA5 peak of the sLCA5-ΔVQT complex contained two fused peaks. The double LCA5 peak showed two different molar ratios of LCA5-LC8. The earlier eluted part of the peak had a molar ratio of 2:1 which is the same as we measured in the ITC experiment, but the later part of the peak showed a 1:1 molar ratio corresponding to a saturated complex (Fig. 6c).

Although LCA5 showed a slight propensity for higher order oligomerization, the oligomeric void volume fraction formation heavily depends on the presence of LC8, the number of binding sites and the K_d_ of the presented motifs. Compared to the wild type, the absence of the weaker CQS containing motif, in the case of the sLCA5-ΔCQS complex, resulted in a decreased oligomerization ability. However, the loss of the stronger VQT-containing motif nearly abolish the oligomerization ability of the LCA5.

### LCA5 forms spherical oligomeric complexes of 30–40 nm size

Based on our gel filtration experiments, LCA5 is able to form oligomers, and the amount of this oligomeric fraction is highly proportional to the presented LC8 binding sites, and the dissociation constant of the presented motifs. To characterize the oligomeric complexes formed by sLCA5-WT and sLCA5-ΔCQS in the presence of LC8, we performed negative staining transmission electron microscopy (TEM) experiments.

The negative staining of sLCA5-WT-LC8 and sLCA5-ΔCQS-LC8 complexes revealed that these variants are able to form spherical objects in vitro in the 30–40 nm size range (Fig. 6f–i). The size distribution and the amount of the formed spherical complexes however differ between the wild type and the sLCA5-ΔCQS variant. Although the oligomeric complexes of the sLCA5-WT and sLCA5-ΔCQS variants show uniform size distributions but comparing the two populations of complexes a significant 20% increase was detected in case of the wild-type complex with a measured diameter median of ~ 39 nm (Fig. 6j). The measured diameter median of the sLCA5-ΔCQS complexes was 32 nm and moreover, the amount of detectable spherical objects was also decreased (Fig. 6f, h).

In the case of the sLCA5-ΔVQT-LC8 complex, the amount of detectable oligomeric particles was negligible compared to the wild type and the sLCA5-ΔCQS variant. The single mutant sLCA5 variants also show negligible potential to form oligomeric particles. We only detect a notable amount of particles around the edges of the grids in case of the sLCA5-ΔCQS variant, but the size distribution and the shape of these particles varied around a much wider distribution, and the particles were mostly stuck together in a more aggregation-like manner and might also be artifacts of sample preparation.

## Discussion

In this work we characterized the interaction of LCA5 protein with LC8. LC8 is a hub protein that was suggested to promote the dimerization of partner proteins. LCA5 is the product of the known ciliopathy gene, whose mutations cause the degradation of photoreceptor cells in Leber congenital amaurosis, however, it is expressed in a wide range of cell types^31^. Here, we showed that the two proteins colocalize in multiple cellular locations in a cell-cycle-dependent manner, and indicate that the interaction between LCA5 and LC8 is not restricted to the ciliary basal body, but is also present in the centrosome. Previous results showed that LCA5 and LC8 are involved in a physical association^33^. In this work, we verified the direct interaction between the two proteins and revealed interesting features of their interactions.

We showed that the binding of LCA5 to LC8 is mediated through two short linear motifs. One of these motifs contains a VQT triplet that largely complies with the standard LC8 binding motif consensus and was characterized by a high score in our motif filter protocol^10^. This motif was validated at the peptide level with a low micromolar K_d_ and showed a sub-micromolar binding affinity at the protein level. However, LCA5 contains an additional motif with a CQS tripeptide that significantly deviates from the consensus and was originally not recognized as an LC8 binding motif. The interaction of the CQS motif with LC8 could not be verified at the peptide level either. In contrast, ITC measurements revealed that the CQS containing motif can bind LC8 with a low micromolar affinity. In addition, mutation experiments indicated that the two binding sites behave very similarly in the context of a larger protein fragment, and both of them need to be mutated to abolish binding to LC8. Altogether these results underlie the importance of the context in linear motif-based interactions and the need to rule out both false positives and false negatives when characterizing binding motifs^34^.

Our CD measurements showed that LCA5 had a large helical content and was capable of forming coiled coil regions on its own, without LC8. However, the coiled coil formation created an avidity effect, commonly observed for LC8 partners^11,30,35^ and enhanced the affinity for the interaction with LC8. This enabled the binding of the LCA5 fragment containing only the weaker motif with the first motif mutated. The coiled coil structures were partially stable at physiological temperature. Binding of LC8 did not increase the coiled coil content of LCA5 notably but increased its thermal stability, and for this stabilization effect both binding motifs were required. ITC measurement and the densitometric analysis of the gel filtration experiment suggested that the minimal oligomeric form of LCA5 is tetrameric.

The stoichiometries measured by ITC indicated that LC8 interconnects the LCA5 units without occupying all the available binding sites, and forms non-saturated complexes with a multivalent promiscuous binding mode. In agreement with this, gel filtration showed the presence of low oligomeric fractions of the sLCA5 variants that formed non-saturated complexes. These low level oligomeric complexes showed a wide range of composition depending on the LC8 binding motifs present. The wild type LCA5 showed a spectrum of non-saturated “intermediate” oligomeric forms from the basic tetrameric complex to higher order oligomers near void volume size. In case of the single mutants, the only detected non-saturated oligomers were tetramers. However, sLCA5-ΔVQT construct contained both saturated and unsaturated forms in low oligomeric state.

Interestingly, the gel filtration study revealed the formation of higher order oligomers. The higher order oligomer formation was present to a small extent even in the case of the double mutant of LCA5 which could not bind LC8. However, this tendency significantly increased, with the addition of LC8, largely proportionally with the number of LC8 binding sites and over time. In the higher order oligomeric fraction, the binding sites became saturated. The wild-type LCA5 had the most pronounced tendency to form higher order oligomers, while the sLCA5-ΔCQS, single mutant with the stronger motif, showed a decreased oligomerization propensity. The sLCA5-ΔVQT construct had very little oligomerization ability, similarly to the ones without LC8. As revealed by TEM, the higher order oligomers formed spherical particles with a diameter of 30–40 nm. The number and size distribution of particles, in agreement to our gel filtration studies, was proportional to the ability of the sLCA5 variants to form higher order oligomers.

Altogether, our results highlight a possible new, important function of LC8, by driving the oligomerization of partners that already formed low-oligomeric forms. In contrast to the general dimerization engine role^6^, LC8 is not required for direct dimerization of LCA5, but it enhances its further oligomerization. In this regard LCA5 shows similarities to NEK9^19^. In the case of LCA5, however, LC8 drives the formation of a dynamic range of oligomers involving unsaturated low-order oligomers as well as higher order oligomers.

**Figure 7:**
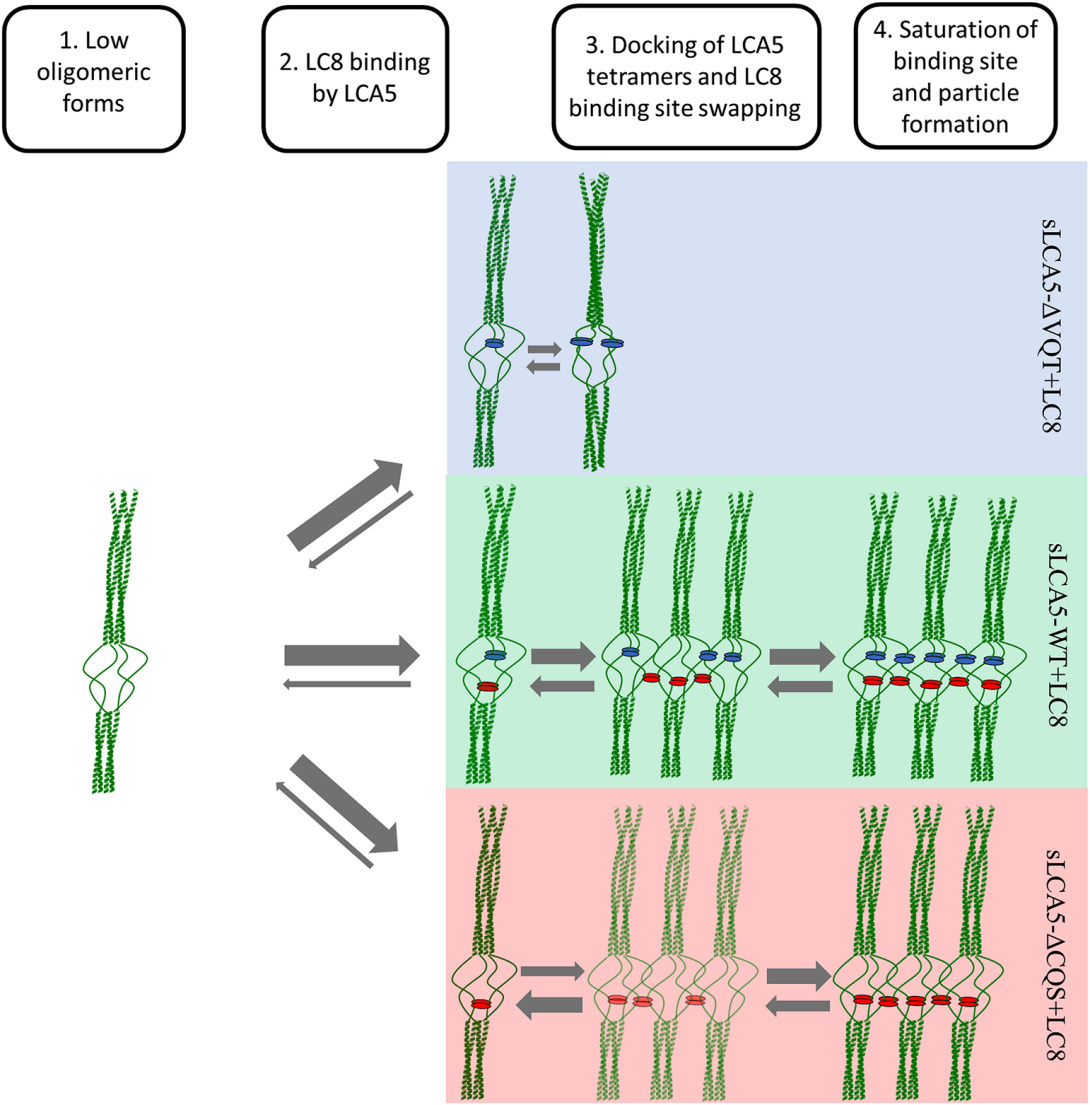
LC8 dependent oligomerization model of LCA5. An initial low oligomeric state is possibly the native form of LCA5 without the binding of LC8. The addition of LC8 to the single mutants and to the wild type initiated an oligomerization process dependent on the affinity of the presented linear motifs and the number of binding sites. The sLCA5-ΔCQS-ΔVQT form, lacking any LC8 binding sites, mostly remains in the initial low oligomeric state. In case of sLCA5-ΔVQT, the low affinity CQS motif is able to bind LC8, but the equilibrium of complex formation is likely shifted towards the formation of saturated tetrameric complexes. The higher affinity motif containing sLCA5-ΔCQS mutant is able to form higher order oligomers by swapping the intra-tetrameric crosslinks to inter-tetrameric ones, but the one presented motif is not enough to stabilize the intermediate forms in a detectable amount (faded intermediate form)*.* The wild type sLCA5-WT could form higher order oligomers in a more effective way in accordance with its increased crosslinking potential compared to the single mutant constructs and forms stable complexes in the range of tetramers to higher order oligomers. The blue spheres represent the N-terminal low affinity motif binding LC8 molecules, the red spheres the higher affinity ones. The thickness of the arrows are proportional to the shifting of the equilibrium of complex formation.

We present here a theoretical model of the LC8 driven non-stoichiometric oligomerization mode of the LCA5 (Fig. 7). In the absence of LC8, the native LCA5 population is likely to be in a tetrameric form, with a very small tendency to form higher order oligomers which purely relies on coiled coil interactions. The addition of LC8 to the system shifts the equilibrium towards the formation of higher order oligomers by stabilizing the coiled coil interactions of the intermediate forms. The formation of these intermediate complexes could only be realized between non-saturated LCA5 tetramers. The non-saturated state provides the excess of LC8 binding sites enabling a dynamic (fuzzy) binding mechanism, where the LC8 molecules could jump between binding sites and could drive the formation of non-saturated intermediate forms. As a result, the dimeric interactions with LC8 could be formed within the same tetramer but could also be formed between tetramers, stabilizing the transiently formed coiled coil interactions. Over time, more and more LC8 dimers could dock into the newly formed intermediate complexes and drive them to the formation of saturated higher order oligomers. This oligomerization model heavily relies on the presence of the two LC8 binding motifs. The importance of the weaker motif is demonstrated by the decreased oligomerization ability of the sLCA5-ΔCQS complex due to the fewer possible crosslinks. This type of intermediates are more likely to dissociate to unsaturated tetramers. In contrast, the absence of a strong motif resulted in an impaired ability to form large intermediate complexes. Over time, this leads to the stabilization and saturation of the tetramers, which become the dominant saturated form instead of higher order oligomers. This two-faceted behavior of the two distinct motifs can lead to a coexistence between the non-saturated low level oligomers and the higher order oligomers when both of the binding motifs are present. The coexistence of the wide range of stable saturated and non-saturated oligomeric forms could be the basis of the fine-tuning of oligomerization status of LCA5 (Fig. 7).

Oligomerization is widespread in the centrosomal system. Many centrosomal proteins lack ordered globular domains and are predicted to contain disordered regions, as well as large coiled coil segments. These proteins can assemble into layers and bundles that are essential for maintaining the structural integrity and functionality of the pericentriolar matrix. Interactions through coiled coil regions are typical in the case of large centrosomal proteins^36–39^. However, additional factors could also play an important role in the organization of this dense protein network whose material state is under considerable debate^40^ Here, we show that LC8 interacts with LCA5 and drives its oligomerization. Interestingly, LCA5 also interacts with Oral-facial-digital syndrome 1 protein (OFD1), a central player in the centrosome-ciliary system^41,42^. OFD1 was also shown to contain an LC8 binding motif^11^. Our preliminary results indicate that LC8 interacts with several other proteins in this ciliary-centrosome system that share the sequence features of LCA5 (manuscript in preparation). This suggests that the oligomerization model presented here could be more widespread among centrosomal proteins and highlights LC8 as a novel essential component of the centrosome organization.

In conclusion, we characterized the interaction between LCA5 and LC8 and identified the specific binding motifs recognized by LC8. The methodology we followed during the experimental process highlighted the importance of weak non-canonical motifs which could be missed at the peptide level, nevertheless are important for the function at the protein level. Based on the complex interplay of structure formation and oligomerization of LCA5, we introduced a new binding model for LC8 related interactions. The novel oligomerization engine function of the LC8 proposed here suggests that LC8 could be essential for the structural organization of the centrosomal system. Further details of LC8 binding partners in the centrosomal system could reveal more insights into the organization and function of this complex and important system, which could facilitate the deeper understanding of the molecular basis of various types of ciliopathies.

## Materials and methods

### Plasmid constructs

Full-length and truncated (96–430) coding sequences of LCA5, were obtained by PCR from cDNA library isolated from HEK293 cell line. The full-length LCA5 sequence was inserted into a pcDNA-3.1 + vector containing an N-terminal FLAG-tag followed by a linker corresponding to the GGSG sequence. The LCA5^96–430^ sequence was inserted into the in-lab modified bacterial expression vector pET-MBP (containing an N-terminal MBP-tag). The sLCA5-ΔVQT, -ΔCQS, and -ΔCQS-ΔVQT mutants were generated by quick-change mutagenesis. pEV vector including LC8 (DYNLL2) with an N-terminal 6xHis-tag was a generous gift of Péter Rapali and László Nyitray. For the pull-down experiments, DYNLL2 was cloned to pETARA vector (modified pET vector, with N-terminal GST-tag). For co-immunoprecipitation full-length LC8 (DYNLL2) was cloned into an N-terminal HA-tag followed by a short GGSG linker containing pcDNA3.1 + vector.

### Cell culture and transfection

HEK-293 (ATCC CRL-1573) and hTERT-RPE1 (ATCC CRL-4000) was the generous gift of Dr László Nyitray and Dr András Málnási-Csízmadia respectively. Cells were cultured in DMEM medium (Lonza) supplemented with 10% fetal bovine serum (Lonza) and 100 μM streptomycin-penicillin (Lonza) in a 5% CO_2_ atmosphere at 37 °C. The cells were transfected with Turbofect (Thermo Fisher Scientific) according to the manufacturer’s protocol.

### Co-immunoprecipitation and immunoblotting

HEK-293 cells were transfected with FLAG-LCA5-pcDNA3.1 + and HA-LC8-pcDNA3.1 + constructs and incubated for 48 h. After 48 h, cells were washed with ice-cold DPBS (Lonza) and lysed on ice with lysis buffer (50 mM TRIS 2 mM EDTA 100 mM NaCl 10% Glycerol and 0.5% IGEPAL supplemented with protease (Sigma Aldrich) and phosphatase (Sigma-Aldrich) inhibitors). Total protein concentration was determined by the BCA kit (Sigma Aldrich). For each co-immunoprecipitation reaction 1 mg total protein was used and 20 μg of anti-lebercilin (19,333-1-AP Proteintech), anti-LC8 (16,811–1-AP Proteintech), polyclonal antibody, anti-FLAG (8146 Cell Signaling Technologies), anti-MYC (2276 Cell Signaling Technologies) or anti-HA (sc-7392 Santa Cruz Biotechnology) monoclonal antibody was added respectively and incubated for 4 h at 4 ºC. The immunocomplexes were incubated with protein A/G sepharose beads (Cell Signaling Technologies) overnight. The immunocomplex-containing beads were washed three times in lysis buffer, then two times in detergent-free lysis buffer, proteins were eluted by boiling in 2 × Laemmli buffer. The samples were analyzed by 10% tricine-SDS PAGE. The SDS gel was cut half above the 35 kDa marker band and subsequently blotted onto two separate Amersham Hybond PVDF membrane (GE Healthcare). The LCA5 containing upper half was blotted for 180 min, the LC8 containing lower part was blotted for 80 min. The membranes were blocked in 5% BSA and the LCA5 and LC8 containing parts are incubated with mouse anti-FLAG 1:1000 (8146 Cell Signaling Technologies), and rabbit anti-LC8 1:1000 (ab51603 Abcam) primary antibodies respectively overnight at 4 °C. After washing with TBS-T, membranes were incubated with secondary antibodies (ECL PLEX Goat-Anti-Rabbit IgG CY5 1:1500 and CL PLEX Goat-Anti-Mouse IgG CY3 1:1500, Ge Healthcare) for 1 h at room temperature, then scanned in a Typhoon scanner (GE Healthcare).

### Protein expression and purification

6xHis-tagged LC8 and N-terminal MBP, C-terminal 6xHis-tag-fused LCA5 variants were expressed in E. coli BL21 (DE3) Rosetta cells (Novagene). Cultures were grown in LB-media, supplemented with 100 μg/ml ampicillin, 30 μg/ml chloramphenicol, and in case of MBP-fused LCA5 variants 2 g/l glucose at 37 °C until an optical density of 2.5–3 McFarland units was reached. Overnight expression at 18 °C was induced by 0.1–0.3 mM IPTG. LC8 was purified using Ni Profinity IMAC Resin (BioRad) followed by anion-exchange chromatography on HiTrap Q-HP column (GE Healthcare), and a final step of size-exclusion chromatography on Superdex 75 10/300 column (GE Healthcare). LCA5 variants were purified using amylose resin (New England Biolabs), followed by IMAC, as described above.

### Isothermal titration calorimetry

LC8 and MBP-fused LCA5 constructs were prepared in PBS supplemented with 1 mM TCEP. Before measurement, protein samples were dialyzed overnight in PBS pH 7.4 (Orange Scientific, OrDial D14 membrane). Peptides were diluted from stock with the buffer used for dialysis. The concentration of samples was determined based on absorbance at 280 nm in the case of proteins, and 205 nm for the peptide. Experiments were performed using MicroCal PEAQ-ITC (Malvern Panalytical), LCA5 peptides were measured at 25 °C, proteins were analyzed at 37 °C. Lebercilin peptide was injected into the cell 18 times 2 μl volumes, with 5 min timing between injections. In case of the protein, LCA5 was placed in the sample cell, and LC8 was injected during 37 injections, 1 μl each. The results were evaluated using MicroCal PEAQ-ITC Analysis Software.

### Fluorescence polarization

Fluorescence polarization experiments were carried out at 25 °C in PBS supplemented by 2 mM TCEP and 0.05% Brij using a Synergy H4 (BioTek Instruments) plate reader. N-terminally fluorescein 5-isothiocyanate (FITC) labeled, known binding peptide of LC8, BMF (TSQEDKATQTL) was used as a reporter peptide to form LC8-BMF complex and LCA5 was applied to compete the BMF peptide. Briefly, 50 nM labeled BMF peptide was mixed with LC8 in a concentration to achieve 80% saturation, determined by direct titration (12 μM at 80% saturation). Subsequently, the above-mentioned complex was titrated with a two-fold dilution series of competitor LCA5 peptide (DLCTKGVQTME) (start at 500 μM) and the decrease of polarization was recorded. To determine the K_d_ of the LCA5-peptide-LC8 complex, first the K_d_ of the BMF-LC8 complex was calculated. The polarization signal recorded at direct titration of BMF peptide with LC8 was plotted against the concentration of LC8. Then the K_d_ of the BMF-LC8 complex was calculated by the fitting of a direct binding equation in Origin 8. To determinate the K_d_ of the competitor peptide, the measured polarization was plotted against the concentration of LCA5 peptide in the competitive titration and the data was fitted by the competitive binding equation in Origin 8 where K_d_ of the BMF-LC8 complex was one of the parameters of the competitive equation. The titrations were carried out in triplicates in a 384 well plate format.

### GST pull-down experiments

For the pull-down experiments, GST-fused LC8 was used as bait, and MBP-, 6x-His-fused LCA5 variants as prey. For controls, GST and MBP were used. Proteins were expressed in 100 ml cultures of E. coli BL21 (DE3) Rosetta cells as described above. Cells were harvested, resuspended in 5 ml PBS supplemented with 1 mg/ml lysozyme and 1 mM DTT, sonicated and clarified by centrifugation. 20 μl of Glutathione Sepharose 4B (Macherey–Nagel) (75%) resin was used for each experiment. Baits were incubated with the resin for 1 h at room temperature, then washed with PBS supplemented with 1 mM DTT. Prey proteins were added and incubated for one hour. The beads were washed and eluted by boiling in a 2 × SDS sample buffer and analyzed in tricine SDS page according to the protocol of Schagger et al^43^.

### Immunofluorescence

hTERT-RPE1 cells were seeded on poly-lysine-coated 21 mm coverslips. After 24 h, cells were serum-starved for an additional 72 h. Cells were washed with DPBS (Lonza) two times and once in cytoskeletal buffer (10 mM PIPES, 100 mM NaCl, 3 mM MgCl_2_, 300 mM Sucrose, 5 mM EGTA, 0.5% Triton-X 100, pH 6.9)^44^. Subsequently, cells were fixed in a cytoskeletal buffer containing 4% PFA for 10 min at 37 °C. Fixed cells were washed with DPBS and permeabilised for an additional 30 min in 0.5% Triton-X 100 in DPBS and incubated for 1 h in a blocking buffer (2% BSA, 0.1% Triton-X 100 in DPBS) at room temperature. Primary and secondary antibodies were diluted in a blocking buffer. The cells were incubated with rabbit anti-lebercilin 1:200 (Proteintech 19,333-1-AP), goat anti-LC8 1:100 (Invitrogen PA5-47,957), mouse anti-acetylated-α-tubulin 1:15,000 (Sigma Aldrich 5335S) overnight at 4 °C. Finally, cells were treated with secondary antibodies (anti-goat-Alexa Fluor 488 1:800, anti-mouse-CY3 1:800, anti-rabbit-Alexa Fluor 647 1:800 Jackson ImmunoResearch) for 1 h at room temperature. Coverslips were mounted using ProLong Diamond antifade mountant with DAPI (Invitrogen) and imaged with an inverted Zeiss LSM 800, the image processing was completed with Fiji ImageJ software. The calculations of the colocalization were performed using the JACoP plugin of ImageJ software^45^. The Pearson’s coefficient and the Manders coefficients were calculated on Z-stack images of primary cilia and centrosomes of hTERT-RPE1 cells using manual thresholds. For the statistical analysis of the colocalization a randomization based Costes method was used.

### CD spectroscopy

CD experiments were carried out on a Jasco J-810 spectropolarimeter using a 1 mm quartz cuvette. For the spectra of the individual constructs, 8 scans were recorded and averaged between 200 nm or 190 nm and 260 nm (in continuous scanning mode, data pitch: 0.2 nm, scanning speed: 50 nm/min, response: 2 s, bandwidth: 2 nm). sLCA5-WT and the mutants were measured at 0.1 mg/ml concentration. 1.2 μM LCA5 and 6 μM LC8 were complexed and analyzed. The spectra of LC8 at a concentration of 6 μM was recorded under the same experimental conditions as described before and subtracted from the individual CD spectra of the sLCA5-LC8 complexes. The baseline subtracted data were evaluated using the BeStSel web-server (http://bestsel.elte.hu/index.php)^46,47^. Thermal denaturation experiments were recorded at 222 nm between 10 and 80 °C using 1 °C/min heating rate. The thermal denaturation profile was fitted according to the Gibbs–Helmholtz equation assuming a two-state model as described by Shih et al^48^. After fitting, the curves were normalized by the following equation.

$$y = - \left( {\frac{{m^\circ - A_{n} - T*m_{n} }}{{A_{d} - A_{n} + \left( {m_{d} - m_{n} } \right)T}} - 1} \right)$$

where y is the ratio of native and unfolded state, A_n_ and A_d_ are the initial values of the Gibbs–Helmholtz equation belong to the native and denatured state, m_n_ and m_d_ are the slopes belong to the A_n_ and A_d_ values, m° is the measured CD value in millidegree and T is the temperature in °C.

The calculation of the helical content based on the BeStSel fitted CD spectra, after the subtraction of LC8 spectra and the structural elements of MBP-tag. The MBP-tag secondary structure composition calculation was based on the crystal structure of PDB:1NL5 and calculated in BeStSel. Data evaluation was performed in Origin 8 software.

### Analytical gel filtration

For analytical gel filtration a Superose 6 Increase 10/300 (Cytiva) was used with an effective resolution between 5–5000 kDa range and a void volume above 5000 kDa. The column was equilibrated in DPBS supplemented with 200 μM TCEP. The calibration curve was fitted on the chromatogram of gel filtration standard mix (BioRad) with the addition of Dextran Blue to determine the void volume. 100 μl of LCA5 purified protein or LCA5-LC8 complex was injected at the concentration of 30 μM for LCA5 and 150 μM for LC8, with a flow rate of 500 μl/min immediately or after 60 min of incubation at 37 °C. For densitometric analysis, fractions were collected and 200 μl of samples were precipitated in 1.8 ml 96% ethanol at − 80 °C for 2 h. The samples were centrifugated for 30 min at 14,000 g, and dissolved in 15 μl of SDS sample buffer. 10 μl of each sample was run on Tricine-SDS gel. The calibration of the densitometry was performed on samples of known molar ratio of sLCA5 construct and LC8 (see the original gel used for calibration on Suplementary Fig. S16). Data evaluation was performed by using Origin 8 software and Fiji ImageJ software.

### Transmission electron microscopy

For TEM negative staining experiments, a JEOL JEM-1011 (*JEOL* Ltd., Tokyo, Japan) electron microscope was used. The purified LCA5-LC8 complexes or LC8 or LCA5 forms alone were put together by using LCA5-WT or the single or double mutants at 30 μM and LC8 at 150 μM concentration in a buffer of 20 mM Tris 70 mM NaCl, pH 7.4, and incubated at 37 °C for 1 h. The complexes were diluted to a final 0.2 mg/ml concentration in the buffer and applied to 300 mesh formvar/carbon-coated copper grids (SPI Inc., USA). After 2 min of incubation the surplus was removed by filter paper and 1% uranyl acetate was applied for 1 min as negative staining. Images were taken around the middle of the grid at 5000, 50,000, 150,000 and 300,000-fold magnification. The collected data was analyzed by Fiji ImageJ software, and the statistical analysis was carried out in Origin 8 software.

## Supplementary Information

Supplementary Information.

## Data Availability

All experimental data of the manuscript is available upon e-mail request at Zsuzsanna Dosztányi: zsuzsanna.dosztanyi@ttk.elte.hu.

## References

[1] Ekman D, Light S, Björklund AK, Elofsson A (2006). What properties characterize the hub proteins of the protein-protein interaction network of Saccharomyces cerevisiae?. Genome Biol..

[2] Hu G, Wu Z, Uversky VN, Kurgan L (2017). Functional analysis of human hub proteins and their interactors involved in the intrinsic disorder-enriched interactions. Int. J. Mol. Sci..

[3] Chang X, Xu T, Li Y, Wang K (2013). Dynamic modular architecture of protein-protein interaction networks beyond the dichotomy of ‘date’ and ‘party’ hubs. Sci. Rep..

[4] Liang J, Jaffrey SR, Guo W, Snyder SH, Clardy J (1999). Structure of the PIN/LC8 dimer with a bound peptide. Nat. Struct. Biol..

[5] Singh GP, Ganapathi M, Dash D (2007). Role of intrinsic disorder in transient interactions of hub proteins. Proteins.

[6] Barbar E (2008). Dynein light chain LC8 is a dimerization hub essential in diverse protein networks. Biochemistry.

[7] Barbar E (2001). Dimerization and folding of LC8, a highly conserved light chain of cytoplasmic dynein. Biochemistry.

[8] Rapali P (2011). Directed evolution reveals the binding motif preference of the LC8/DYNLL hub protein and predicts large numbers of novel binders in the human proteome. PLoS ONE.

[9] Rapali P (2011). DYNLL/LC8: A light chain subunit of the dynein motor complex and beyond. FEBS J..

[10] Erdős G (2017). Novel linear motif filtering protocol reveals the role of the LC8 dynein light chain in the Hippo pathway. PLoS Comput. Biol..

[11] Jespersen N (2019). Systematic identification of recognition motifs for the hub protein LC8. Life Sci Alliance.

[12] Singh PK, Weber A, Häcker G (2018). The established and the predicted roles of dynein light chain in the regulation of mitochondrial apoptosis. Cell Cycle.

[13] Pfister KK, Fay RB, Witman GB (1982). Purification and polypeptide composition of dynein ATPases from Chlamydomonas flagella. Cell Motil..

[14] Pfister KK (2006). Genetic analysis of the cytoplasmic dynein subunit families. PLoS Genet..

[15] Makokha M, Hare M, Li M, Hays T, Barbar E (2002). Interactions of cytoplasmic dynein light chains Tctex-1 and LC8 with the intermediate chain IC74. Biochemistry.

[16] Rodríguez-Crespo I (2001). Identi¢cation of novel cellular proteins that bind to the LC8 dynein light chain using a pepscan technique. FEBS Lett..

[17] Kidane AI (2013). Structural features of LC8-induced self-association of swallow. Biochemistry.

[18] Slevin LK, Romes EM, Dandulakis MG, Slep KC (2014). The mechanism of dynein light chain LC8-mediated oligomerization of the Ana2 centriole duplication factor. J. Biol. Chem..

[19] Regué L (2011). DYNLL/LC8 protein controls signal transduction through the Nek9/Nek6 signaling module by regulating Nek6 binding to Nek9. J. Biol. Chem..

[20] Kuhn M, Hyman AA, Beyer A (2014). Coiled-coil proteins facilitated the functional expansion of the centrosome. PLoS Comput. Biol..

[21] Treviño MA, García-Mayoral MF, Jiménez MÁ, Bastolla U, Bruix M (2014). Emergence of structure through protein-protein interactions and pH changes in dually predicted coiled-coil and disordered regions of centrosomal proteins. Biochim. Biophys. Acta.

[22] Dos Santos HG (2013). Structure and non-structure of centrosomal proteins. PLoS ONE.

[23] Woodruff JB (2017). The centrosome is a selective condensate that nucleates microtubules by concentrating Tubulin. Cell.

[24] Doxsey S, McCollum D, Theurkauf W (2005). Centrosomes in cellular regulation. Annu. Rev. Cell Dev. Biol..

[25] Gupta GD (2015). A dynamic protein interaction landscape of the human centrosome-cilium interface. Cell.

[26] Quarantotti V (2019). Centriolar satellites are acentriolar assemblies of centrosomal proteins. EMBO J..

[27] Jakobsen L (2011). Novel asymmetrically localizing components of human centrosomes identified by complementary proteomics methods. EMBO J..

[28] van Dam TJP (2019). CiliaCarta: An integrated and validated compendium of ciliary genes. PLoS ONE.

[29] Andersen JS (2003). Proteomic characterization of the human centrosome by protein correlation profiling. Nature.

[30] Toropova K (2019). Structure of the dynein-2 complex and its assembly with intraflagellar transport trains. Nat. Struct. Mol. Biol..

[31] den Hollander AI (2007). Mutations in LCA5, encoding the ciliary protein lebercilin, cause Leber congenital amaurosis. Nat. Genet..

[32] den Hollander AI, Roepman R, Koenekoop RK, Cremers FPM (2008). Leber congenital amaurosis: Genes, proteins and disease mechanisms. Prog. Retin. Eye Res..

[33] Boldt K (2011). Disruption of intraflagellar protein transport in photoreceptor cilia causes Leber congenital amaurosis in humans and mice. J. Clin. Invest..

[34] Gibson TJ, Dinkel H, Van Roey K, Diella F (2015). Experimental detection of short regulatory motifs in eukaryotic proteins: Tips for good practice as well as for bad. Cell Commun. Signal..

[35] Radnai L (2010). Affinity, avidity, and kinetics of target sequence binding to LC8 dynein light chain isoforms. J. Biol. Chem..

[36] David A (2016). Molecular basis of the STIL coiled coil oligomerization explains its requirement for de-novo formation of centrosomes in mammalian cells. Sci. Rep..

[37] Kim T-S (2019). Molecular architecture of a cylindrical self-assembly at human centrosomes. Nat. Commun..

[38] Fry AM, Sampson J, Shak C, Shackleton S (2017). Recent advances in pericentriolar material organization: Ordered layers and scaffolding gels. F1000Res.

[39] Woodruff JB, Wueseke O, Hyman AA (2014). Pericentriolar material structure and dynamics. Philos. Trans. R Soc. Lond. B Biol. Sci..

[40] Woodruff JB (2021). The material state of centrosomes: Lattice, liquid, or gel?. Curr. Opin. Struct. Biol..

[41] Coene KLM (2009). OFD1 is mutated in X-linked Joubert syndrome and interacts with LCA5-encoded lebercilin. Am. J. Hum. Genet..

[42] Singla V, Romaguera-Ros M, Garcia-Verdugo JM, Reiter JF (2010). Ofd1, a human disease gene, regulates the length and distal structure of centrioles. Dev. Cell.

[43] Schägger H (2006). Tricine-SDS-PAGE. Nat. Protoc..

[44] Hua K, Ferland RJ (2017). Fixation methods can differentially affect ciliary protein immunolabeling. Cilia.

[45] Bolte S, Cordelières FP (2006). A guided tour into subcellular colocalization analysis in light microscopy. J. Microsc..

[46] Micsonai A (2015). Accurate secondary structure prediction and fold recognition for circular dichroism spectroscopy. Proc. Natl. Acad. Sci. U.S.A..

[47] Micsonai A (2022). BeStSel: webserver for secondary structure and fold prediction for protein CD spectroscopy. Nucleic Acids Res..

[48] Shih P, Holland DR, Kirsch JF (1995). Thermal stability determinants of chicken egg-white lysozyme core mutants: Hydrophobicity, packing volume, and conserved buried water molecules. Protein Sci..

