# Supplementary material for: The interaction between LC8 and LCA5 reveals a novel oligomerization function of LC8 in the ciliary-centrosome system
Source: Sci Rep. 2022 Sep 16;12:15623. doi: 10.1038/s41598-022-19454-4 (PMC9481538; doi:10.1038/s41598-022-19454-4)
Supplement: Supplementary file 1 — Supplementary Information. [file 41598_2022_19454_MOESM1_ESM.pdf]

Supplementary Information

The interaction between LC8 and LCA5 reveals a novel oligomerization function of LC8 in the ciliary-centrosome system

Tamás Szaniszló, Máté Fülöp, Mátyás Pajkos, Gábor Erdős, Réka Ágnes Kovács, Henrietta Vadászi, József Kardos, Zsuzsanna Dosztányi

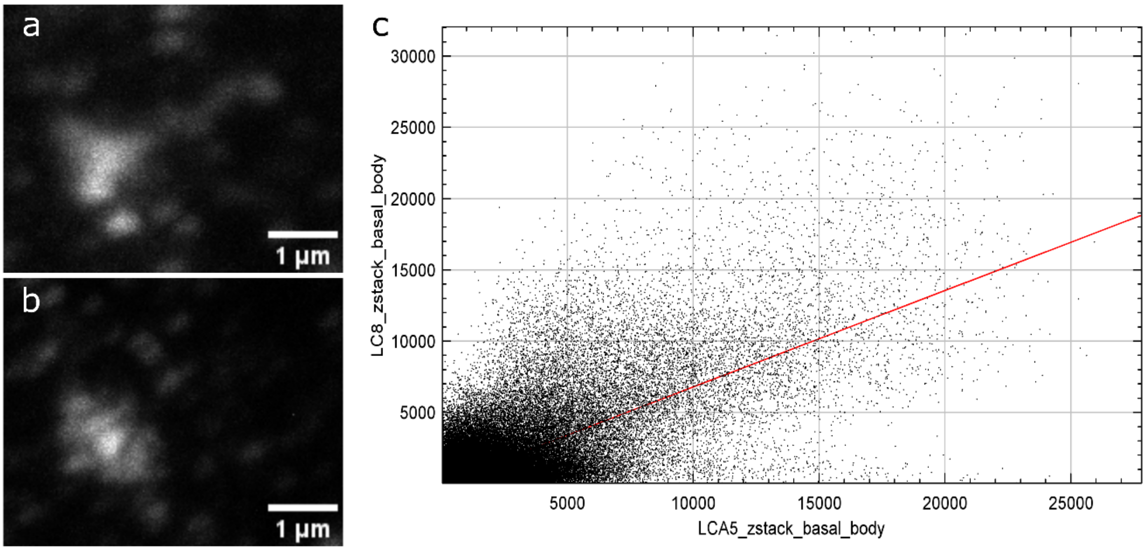

Supplementary Figure S1. Colocalization of LCA5 and LC8 at the ciliary basal body. The LCA5 and LC8 colocalized in the primary cilia, especially in the basal body in hTERT-RPE1 cells. The Pearson's correlation coefficient was 0.715 the thresholded Manders coefficient of the overlap of LCA5 to LC8 was 0.671 and the overlap of LC8 to LCA5 was 0.718. The colocalization was statistically tested against Costes randomization-based colocalization and significantly differs, P=100% (at p=0.05). The calculations were performed on a z-stack image of the primary cilia of an hTERT-RPE1 cell. (a) LCA5 at the primary cilia on max intensity Z-stack projection. (b) LC8 at the ciliary basal body on max intensity Z-stack projection. (c) Cytofluorogram of Z-stack images of the intensities of LCA5 plotted against the intensities of LC8.

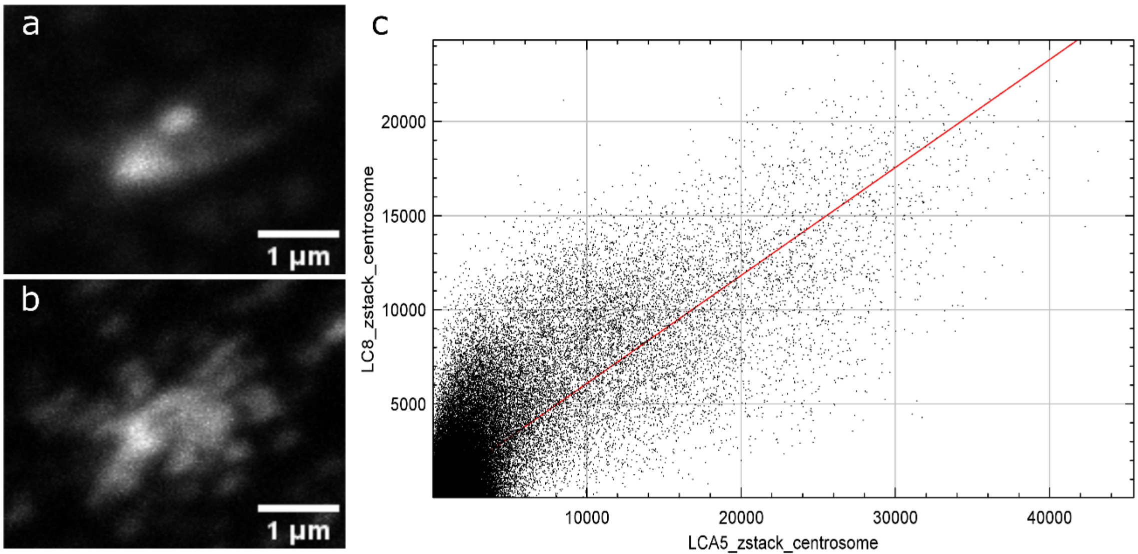

Supplementary figure S2. Colocalization of LCA5 and LC8 at the centrosome. The LCA5 and LC8 colocalized in the centrosome of hTERT-RPE1 cells. The Pearson's correlation coefficient was 0.802 the thresholded Manders coefficient of the overlap of LCA5 to LC8 was 0.929 and the overlap of LC8 to LCA5 was 0.574. The colocalization was statistically tested against Costes randomization-based colocalization and significantly differs, P=100% (at p=0.05). The calculations were performed on a z-stack image of the centrosome of an hTERT-RPE1 cell. (a) LCA5 at the centrosome on max intensity Z-stack projection. (b) LC8 at the centrosome on max intensity Z-stack projection. (c) Cytofluorogram of Z-stack images of the intensities of LCA5 plotted against the intensities of LC8.

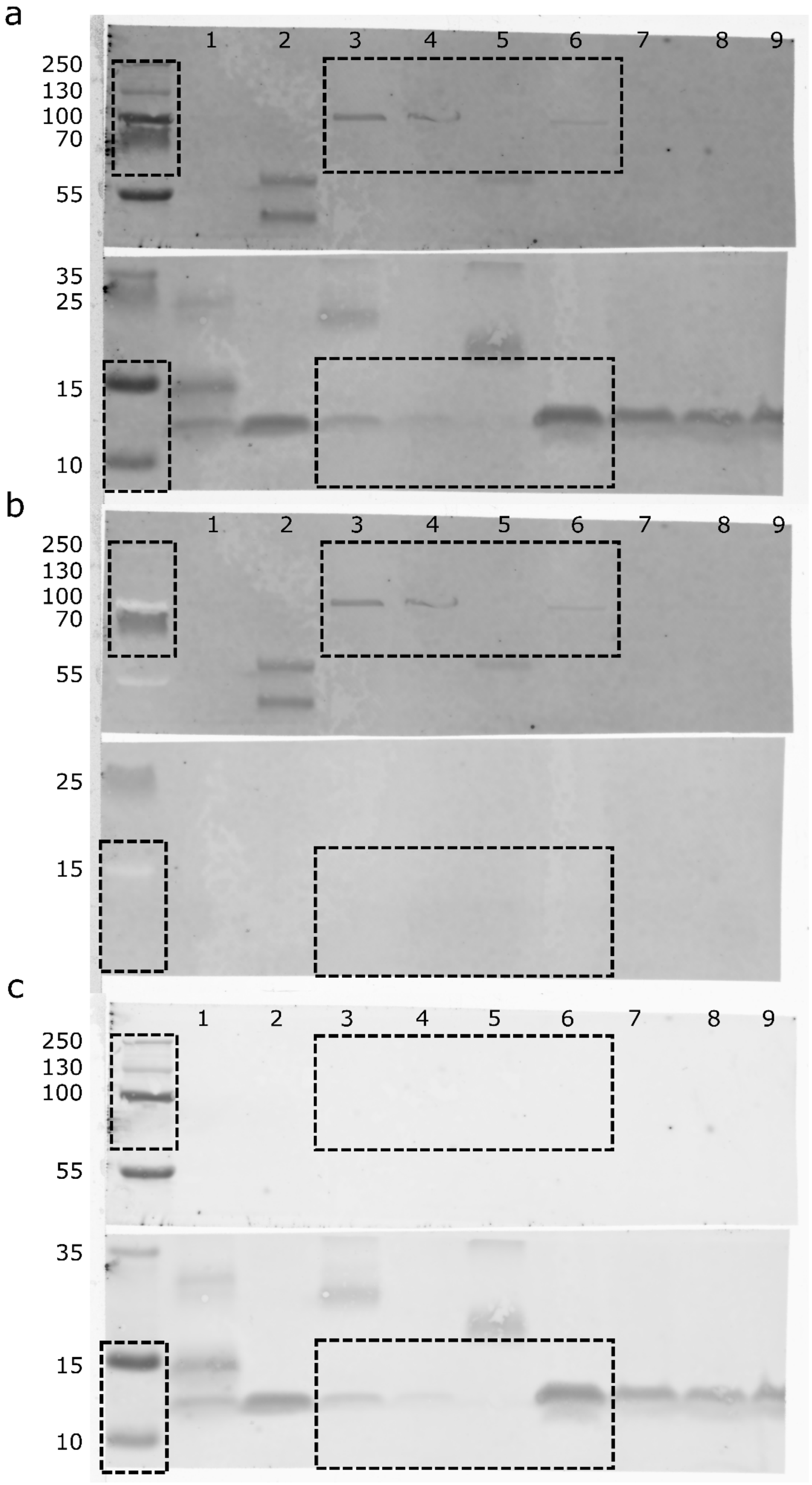
a
1 2 3 4 5 6 7 8 9
250
130
100
70
55
35
25
15
10
b
1 2 3 4 5 6 7 8 9
250
130
100
70
55
25
15
c
1 2 3 4 5 6 7 8 9
250
130
100
55
35
15
10

Supplementary Figure S3. Original immunoblot of LCA5 co-immunoprecipitation. (a) Composite image of the original blot. Hek293 cells were co-transfected with HA-LC8 and FLAG-LCA5. The SDS gel was cut in half and blotted separately, the high mass range (upper part) for 180 min and the low mass range (lower part) for 80 min, as described in the Materials and Methods. Lane 1, immunoprecipitate of the cell lysate using anti DYNLL1 antibody; lane 2, anti-HA antibody; lane 3, anti-LCA5; lane 4, Anti-FLAG; lane 5, for negative control anti-MYC antibody was used to prevent the immunoprecipitation of endogenous LCA5; lane 6, total lysate without immunoprecipitation; lane 7-9, flow-through of anti-MYC, anti-FLAG and anti-LCA5, respectively. Interaction was detected only in the case of anti-LCA5 and anti-FLAG precipitates, using anti-HA and anti-LC8 antibodies no interaction was detected, which might be the result of low expression of FLAG-LCA5 or the competitive binding of antibodies to LC8. PageRuler Plus prestained protein ladder (Thermo Scientific) was used. The dashed line boxes represent the membrane parts used for Figure 1d. **(b)** Original grayscale image of the blot. Cy3 anti-mouse secondary antibody was used and scanned by a 532 nm laser. (**c)** Original grayscale image of the blot. Cy5 anti-rabbit secondary antibody was used and scanned by a 635 nm laser, the lanes and boxes represent the same areas described at the composite image.

a

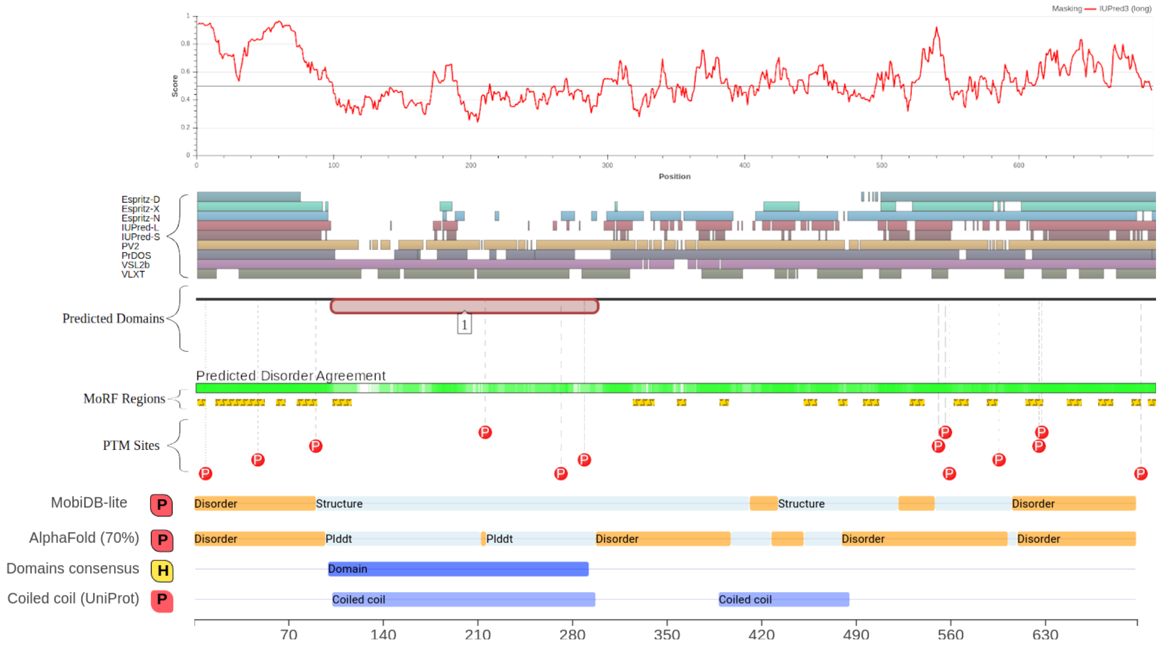

b

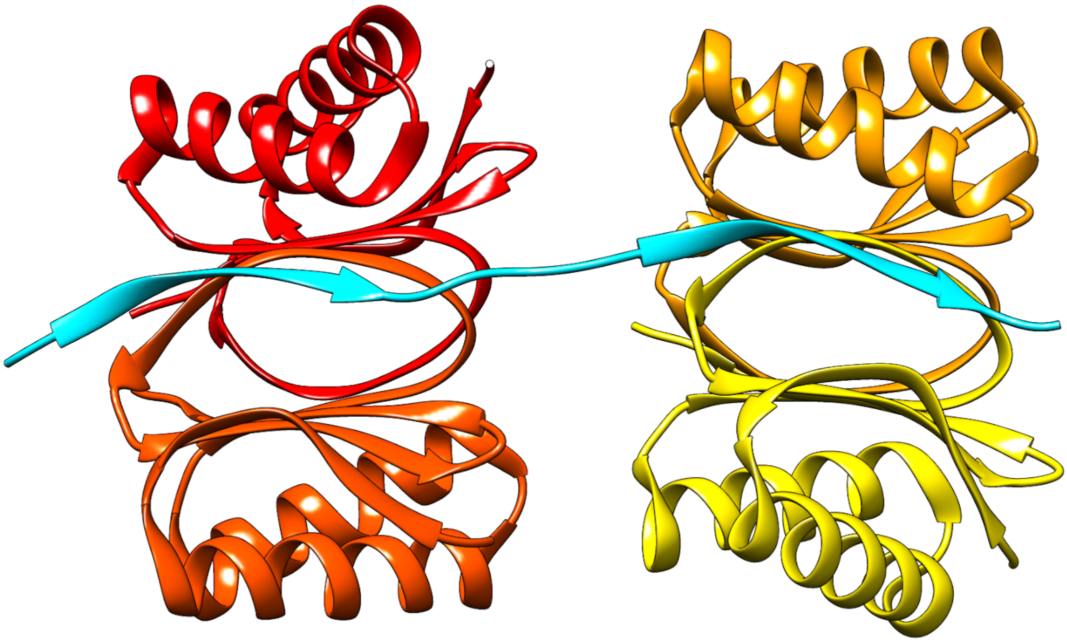

Supplementary Figure S4. Disorder prediction of LCA5 and structural model of LCA5 binding region (a) Disorder prediction profiles of the LCA5 protein. For the IUPred profile no smoothing was applied. The output of the D2P2 platform is also shown (1). We also included MobiDB-lite prediction, AlphaFold pLDDT score, domain consensus, and coiled coil prediction from MobiDB (2,3). With IUPred, regions with scores above 0.5 correspond to disordered regions., and coiled coil regions often show intermediate characteristics with scores around 0.5, as can be observed here. Other methods, MobiDB-Lite or the disordered assessment based on the pLDDT scores of Alphafold2, classify these regions as ordered, while some other methods, such as VSL2b or PV2 predict this region as disordered. However, the structural status of coiled-coil regions cannot be assessed without further

knowledge on their oligomerization state, as these regions are expected to be disordered in monometic state. **(b)** Model of the 314-336 region of LCA5 with 2 LC8 molecules bound to their respective sites based on the structure 4RH7 (4) visualized using UCSF Chimera (5). The model indicates that two binding sites could be simultaneously occupied in theory.

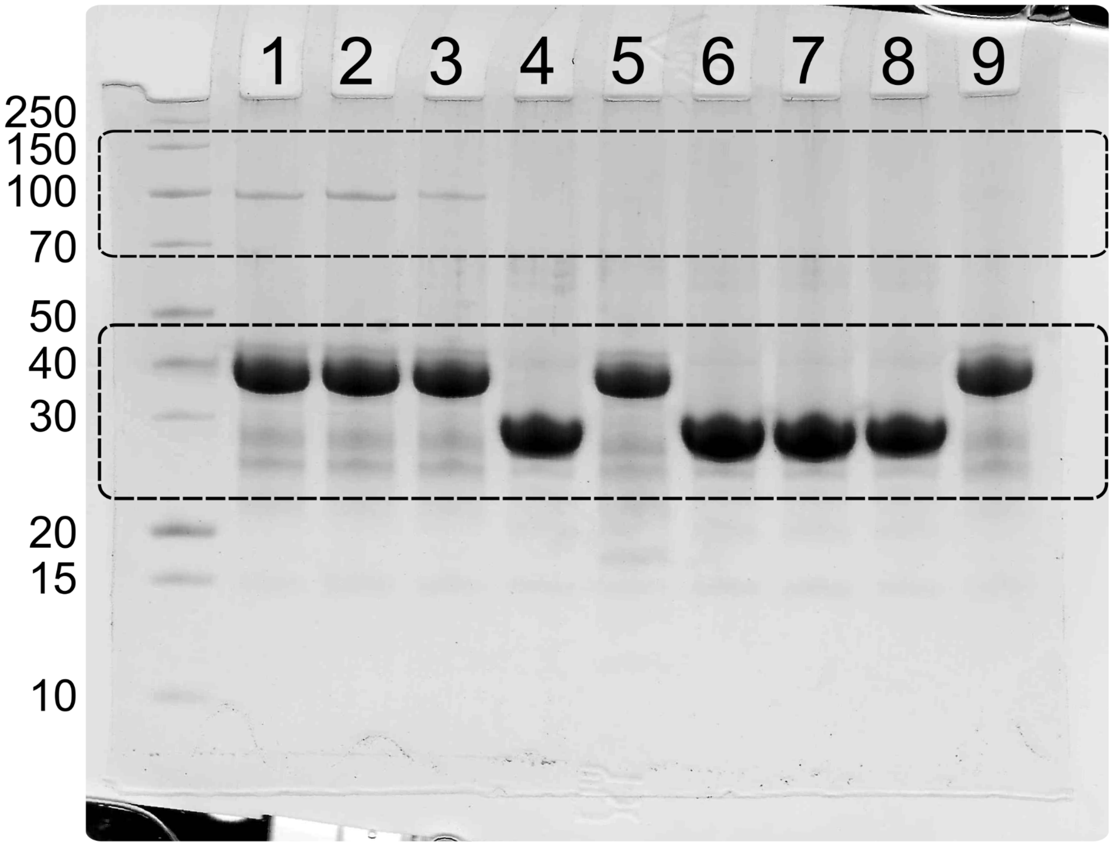

Supplementary Figure S5. Original SDS-PAGE of LC8 GST pull-down. GST-LC8 and GST alone was used as bait protein and sLCA5 constructs and MBP alone was used as prey protein. Lane 1,2,3,5 were sLCA5-WT, sLCA5-ΔVQT, sLCA5-ΔCQS, and sLCA5-ΔCQS-ΔVQT, respectively, pulled down with GST-LC8, lane 4, 6, 7,and 8 were sLCA5-WT, sLCA5-ΔVQT, sLCA5-ΔCQS, and sLCA5-ΔCQS-ΔVQT in pull down reaction with GST alone, and lane 9 was the combination of GST-LC8 and the single MBP. On the left side, the protein marker Page ruler broad range ladder (Thermo Scientific) is provided. The dashed boxes represent the areas cropped and incorporated into Figure 3b.

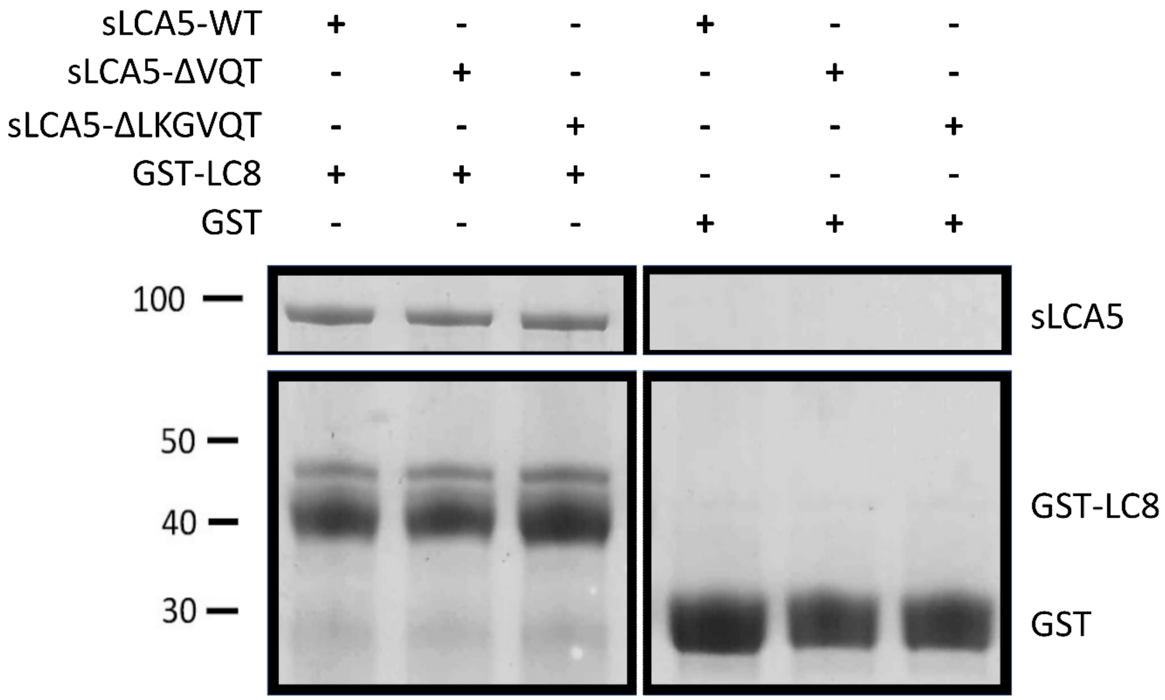

Supplementary Figure S6. GST pull-down experiment of sLCA5-WT, sLCA5-ΔVQT and sLCA5-ΔLKGVQT constructs. The introduced 6 alanine mutations in sLCA5-ΔLKGVQT did not abolish the interaction between LCA5 and LC8. (gel is cropped for better understanding, see the original gel in **Supplementary Fig S5**)

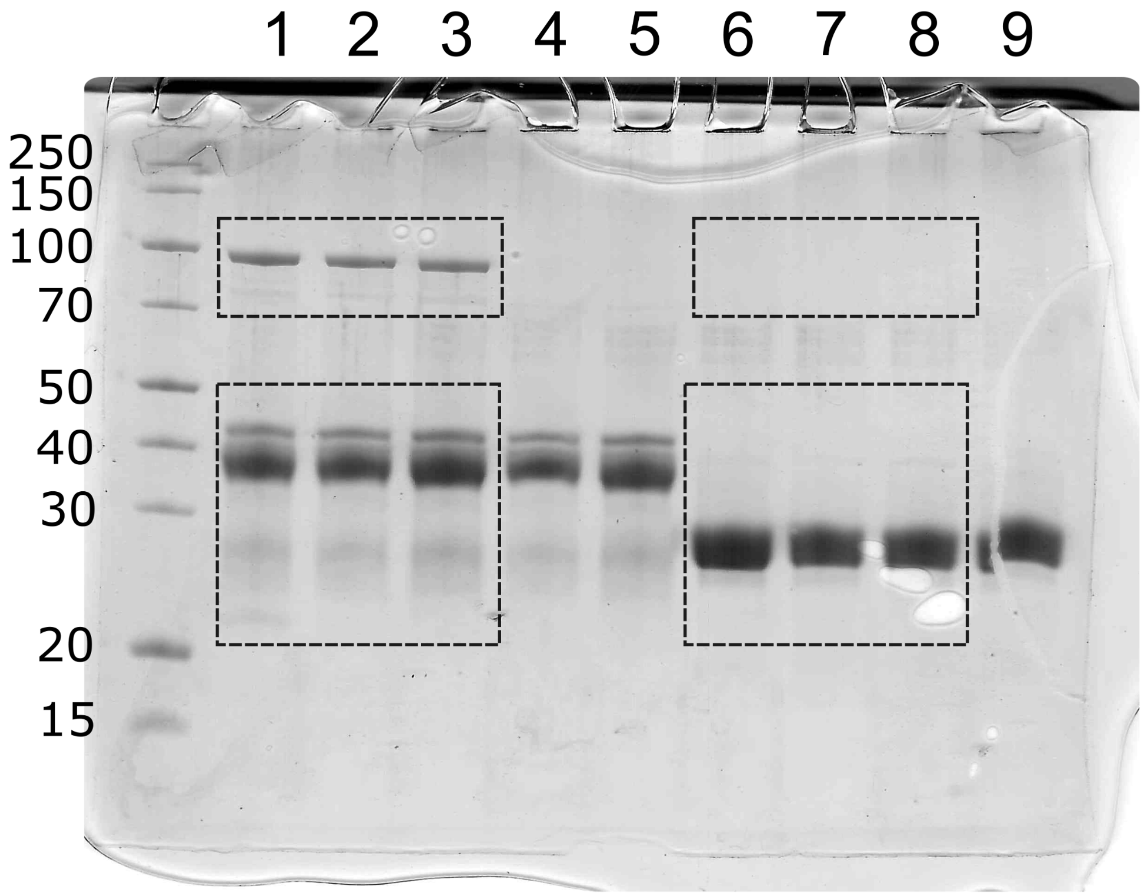

Supplementary Figure S7. Original gel containing the 6 alanine mutant sLCA5-ΔLKGVQT construct. Lane 1-4 were sLCA5-WT, sLCA5-ΔVQT, sLCA5-ΔLKGVQT, sLCA5-ΔCQS-ΔVQT pull downs with GST-LC8, respectively. Lane 5, GST-LC8 alone; lane 6-9, GST alone with sLCA5-WT, sLCA5-ΔVQT, sLCA5-ΔLKGVQT, sLCA5-ΔCQS-ΔVQT, respectively. The very left lane contained the protein marker Page ruler broad range ladder (Thermo Scientific). The dashed boxes represent the cropped areas used for Supplementary Figure S4.

		Composition of complexes		
Gene name	LC8 binding sites	Number of LC8 monomeric binding partners in complex	Number of monomeric LC8 in complex	Ratio of LCA5 monomers to LC8 monomers
SWA (6)	1	2	2	1:1
DYNC1I1 (7)	1	2	2	1:1
WDR34-WDR60 (8)	3	1+1	6	1:3
ANA2 (9)	2	2	4	1:2
ASCIZ (10)	11	2	22	1:11
sLCA5-WT	2	4	4	1:1
sLCA5-single mutants	1	4	2	2:1

Supplementary Table S1. Stoichiometries of various LC8 binding partners in complex with LC8. Known LC8 binding partners according to literature data always form dimers upon LC8 binding. The stoichiometry is basically 1:1 in case of one LC8 binding site, where 2 monomeric partners are dimerized by binding one dimeric LC8. This ratio could increase up to 1:11 in case of ASCIZ where the dimeric ASCIZ possibly could bind 11 LC8 dimers in 11 binding sites, however literature data suggest that these binding sites are not always fully occupied. According to our ITC and CD spectroscopy data, we proposed a very different binding model where the LCA5 forms likely tetramers alone and LC8 could interconnect this dimerized, moreover the possible binding sites are not fully occupied. This resulted in a 2:1 and 1:1 ratio case of single mutant LCA5 construct where one binding site presented and in case of wild type LCA5 where 2 binding sites presented, respectively.

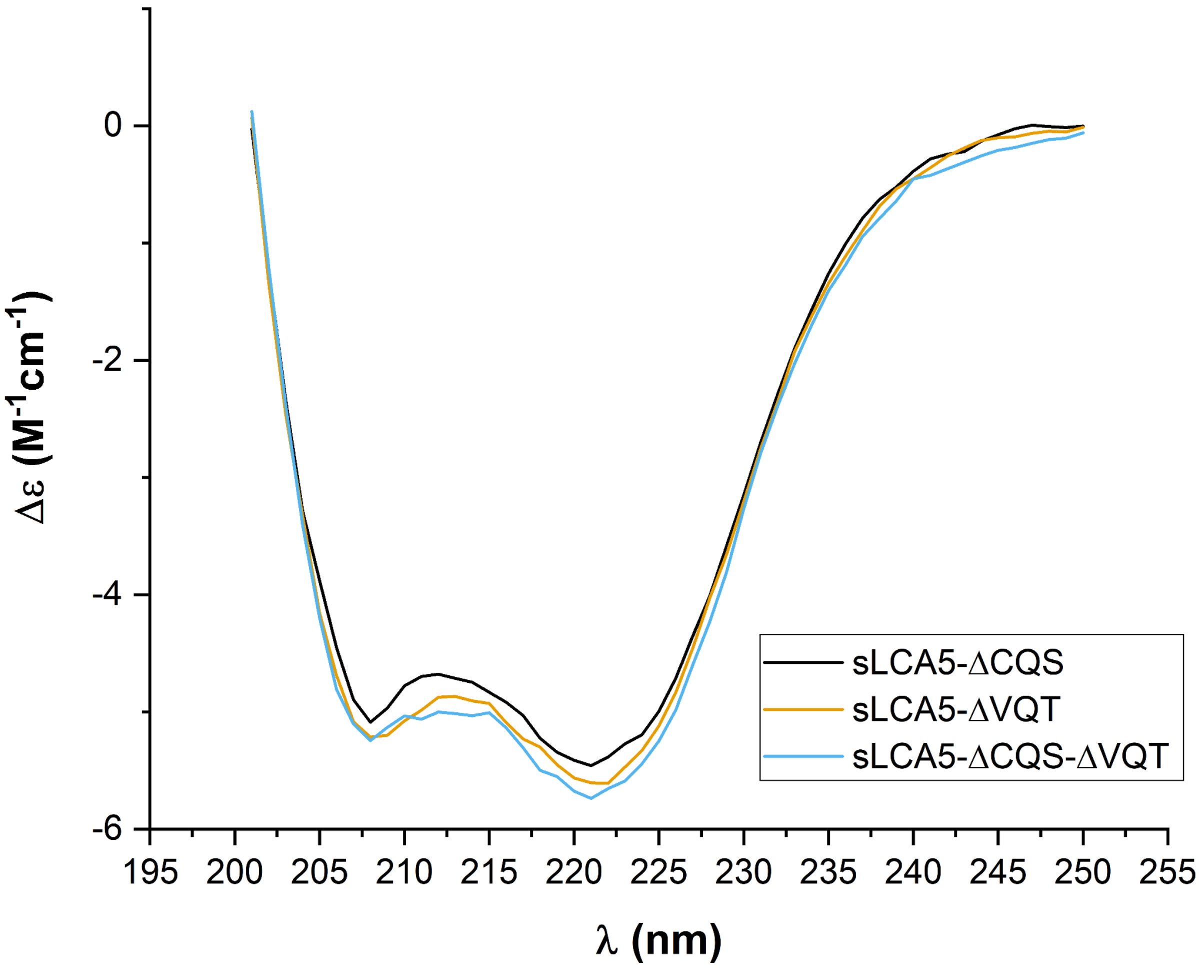

Supplementary Figure S8. Structure determination of sLCA5 mutant constructs by CD spectroscopy. The sLCA5-ΔCQS, sLCA5-ΔVQT and sLCA5-ΔCQS-ΔVQT constructs without LC8 at 20 °C show a coiled coil containing structure based on the ratio between the local minimums of the CD spectra recorded at the 208 and 222 nm. The similarity between the spectra of the various mutant LCA5 constructs indicated that the introduced alanine mutations do not influence significantly the structural compositions of the constructs.

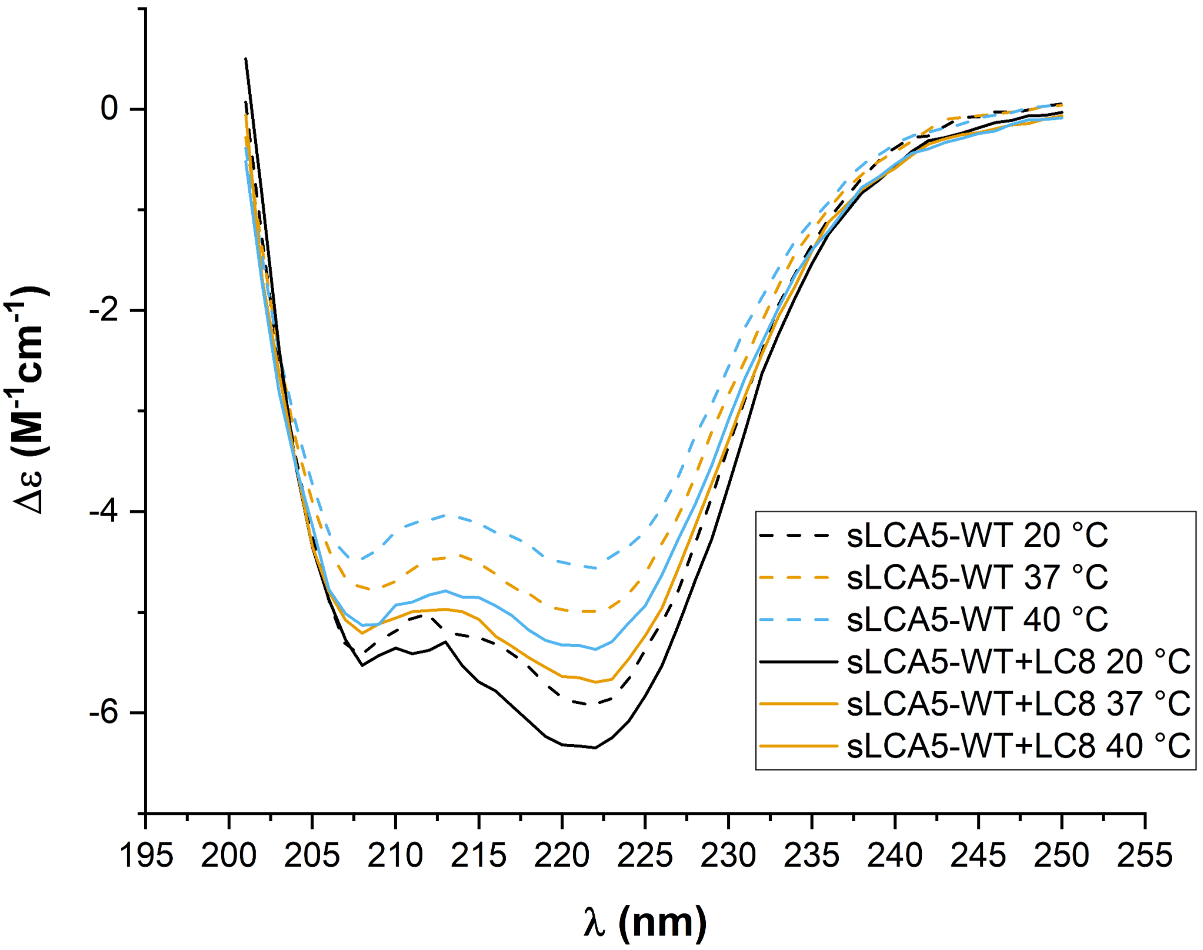

Supplementary Figure S9. CD spectroscopy measurement of sLCA5-WT with and without LC8 between 20 °C and 40 °C. LCA5 complexed with LC8 is able to maintain its coiled coil structure more effectively than LCA5 without LC8 as the temperature rises. The interaction with LC8 possibly could stabilize the coiled coil structure of LCA5. This phenomenon is most pronounced around 40 °C, which corresponds to the melting temperature of the single constructs. In the case of sLCA5-WT without LC8, the amplitude is substantially decreased and the negative minimum at 208 and 222 nm are almost identical, which indicates the destabilization of the coiled coil structures.

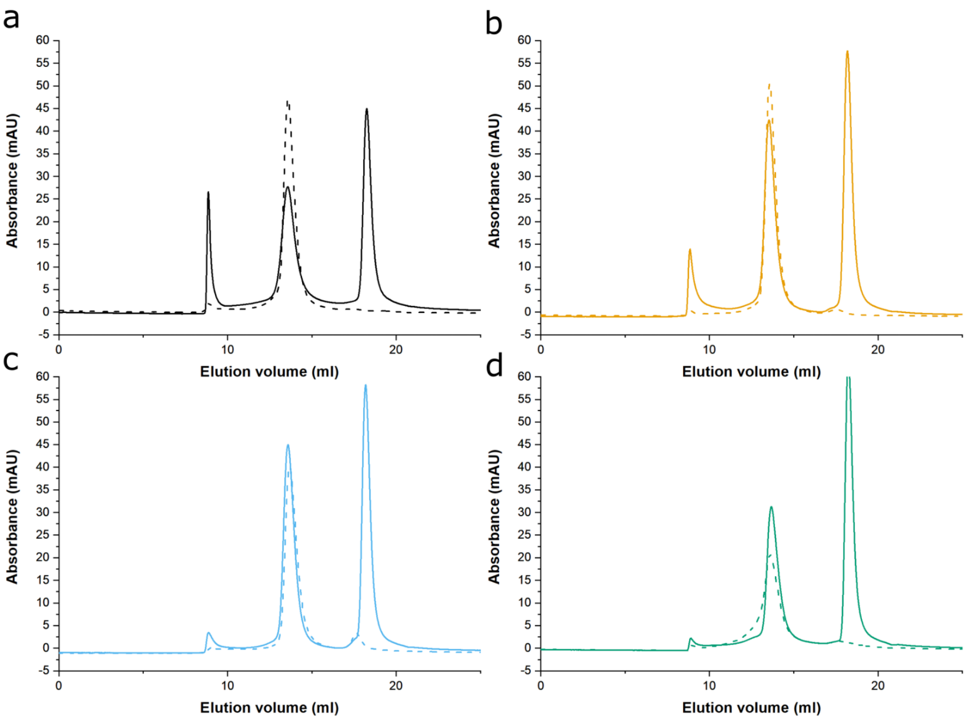

Supplementary Figure S10. Gel filtration of LCA5 variants in complex with LC8 without incubation compared with the single constructs. Analytical size-exclusion chromatograms of LCA5-LC8 complex and single construct injections of (a) sLCA5-WT with LC8 (solid line) and sLCA5-WT (dashed line), (b) sLCA5-ΔCQS with LC8 (solid line) and sLCA5-ΔCQS (dashed line), (c) sLCA5-ΔVQT with LC8 (solid line) sLCA5-ΔVQT (dashed line), and (d) sLCA5-ΔCQS-ΔVQT with LC8 (solid line) and sLCA5-ΔCQS-ΔVQT (dashed line). The LCA5 fractions have the same elution volume in the case of the complexes and the single construct injections, which indicates that LCA5 is in the same low oligomeric form in both cases. According to the ITC experiments, this possibly should be a tetrameric form. The size of the void volume fractions of the wild type and sLCA5-ΔCQS complexes are smaller compared to the complexes after 1 hour of incubation.

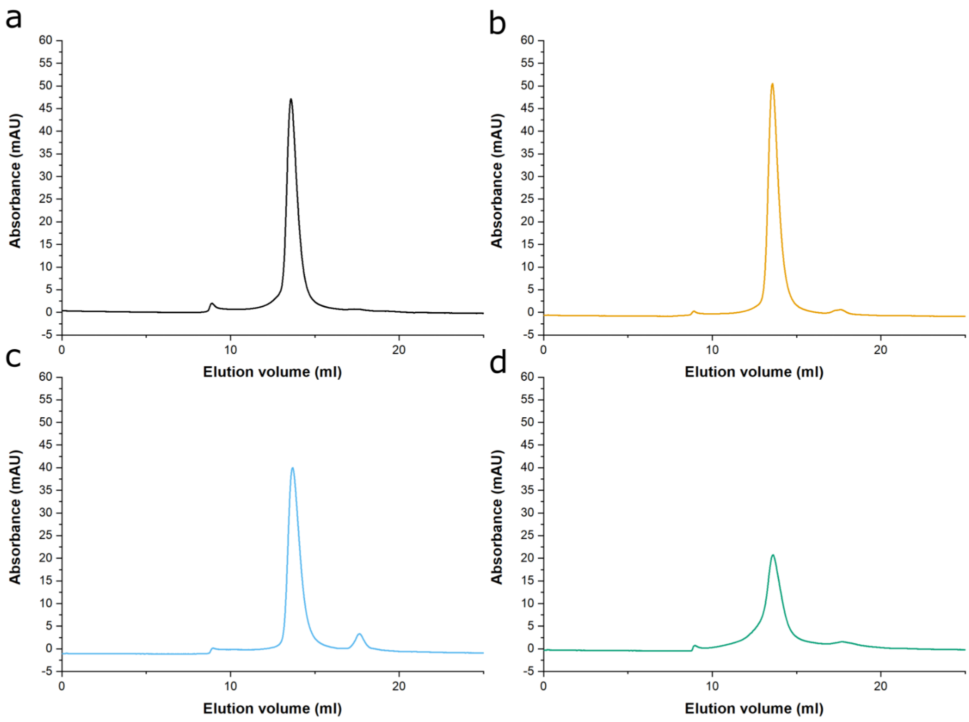

Supplementary Figure S11. Native analytical gel filtration of sLCA5 constructs. Analytical size-exclusion chromatograms of single construct injections of (**a**) sLCA5-WT, (**b**) sLCA5-ΔCQS, (**c**) sLCA5-ΔVQT, and (**d**) sLCA5-ΔCQS-ΔVQT injected without LC8. The single construct injections show minimal propensity to form higher order oligomers in absence of LC8.

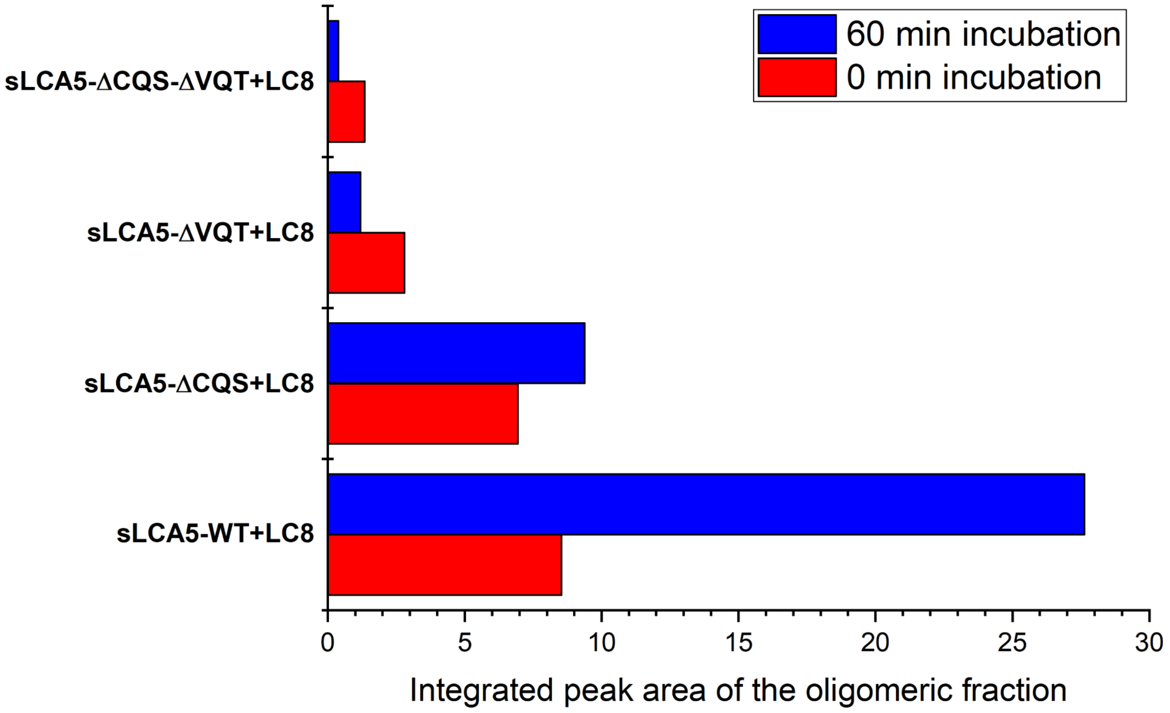

Supplementary Figure S12. Comparison of the void volume fraction sizes of the various sLCA5 constructs in the presence of LC8 and the incubation time dependency of the fraction formation. The void volume fraction of the sLCA5-ΔCQS-ΔVQT and the sLCA5-ΔVQT in the presence of the LC8 was similar in the amount. The different incubation times did not significantly affect the formation of the oligomers. However, the sLCA5-WT and the sLCA5-ΔCQS constructs had a more effective oligomerization potential compared to the sLCA5-ΔCQS-ΔVQT and the sLCA5-ΔVQT forms. The size of the oligomeric void volume fractions of the sLCA5-WT complex without incubation was extended by 30% over the sLCA5-ΔCQS complex under the same conditions. Introducing an extended incubation time, the difference between the two fractions increased by 300 %.

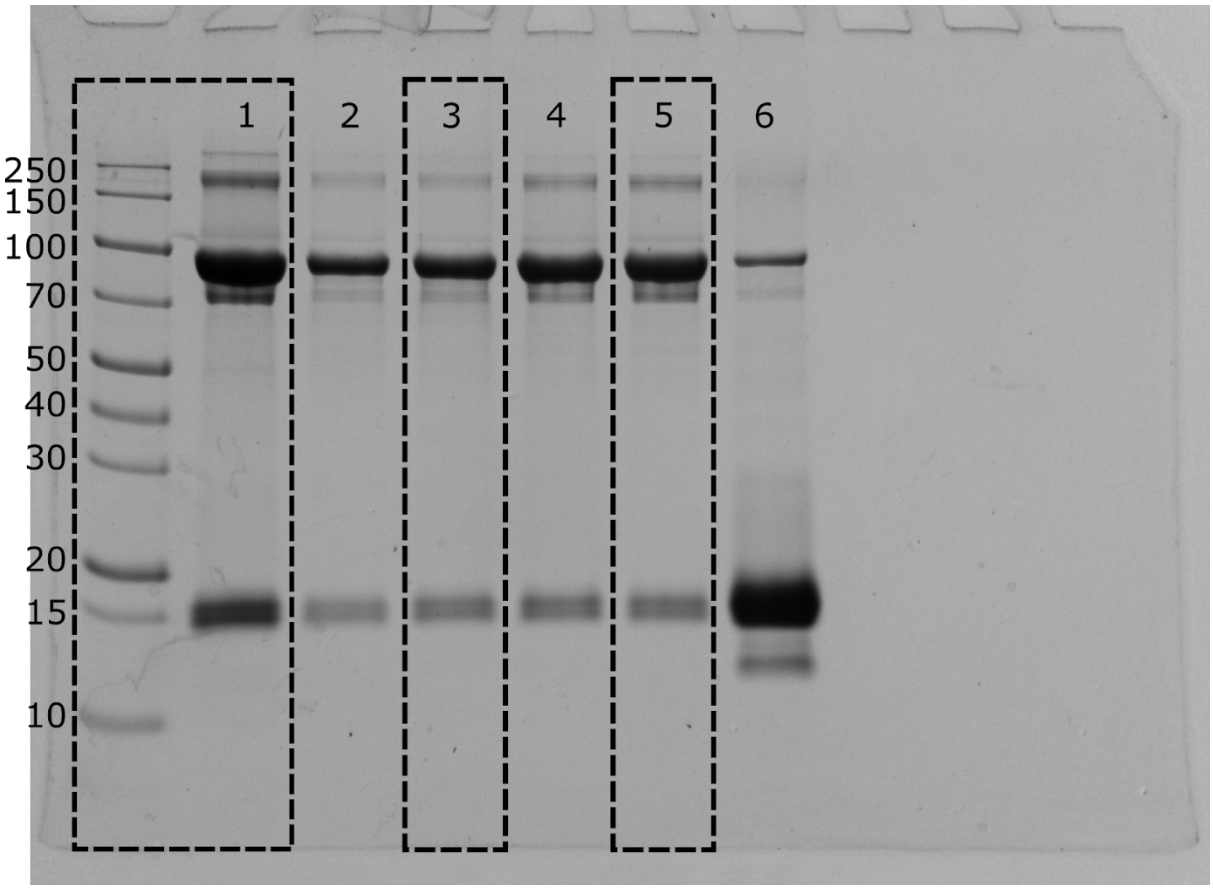

Supplementary Figure S13 Original gel picture of analytical gel filtration fractions of LCA5-WT-LC8 complex. Lane 1 is the oligomeric fraction, and the molar ratio of the LCA5 and the LC8 is 1:2 which indicates a saturated oligomeric complex. Lane 2-3 are the samples from the intermediate fraction with the unsaturated complex molar ratio of 1:1. Lanes 4-5 are the samples of the LCA5 peak fraction with a molar ratio of 1:1. Lane 6 is the sample collected from the LC8 peak. The very first lane is the protein ladder (Thermo Scientific, PageRuler Broad range). The dashed line rectangular markers represent the gel sections used in the densitometric analyses of Figure 6a.

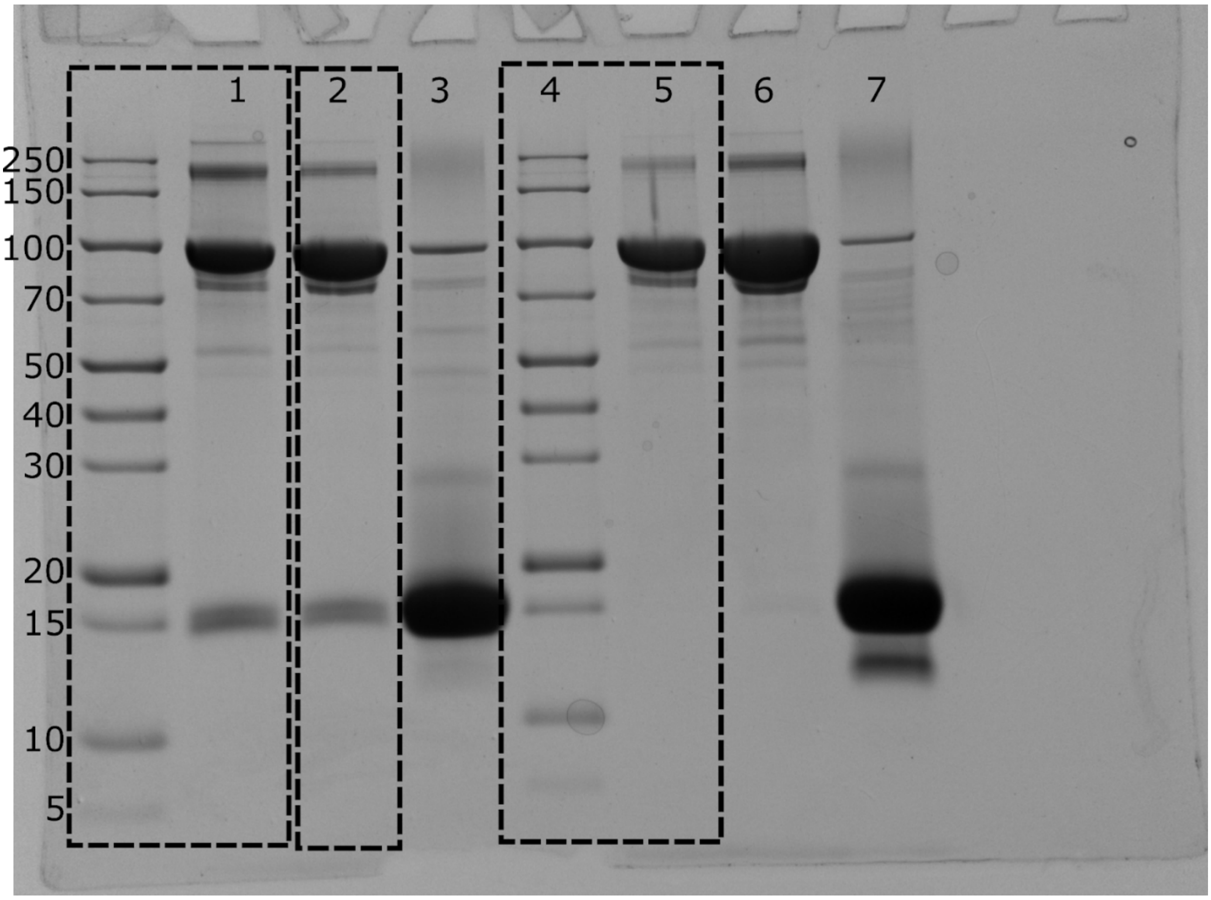

Supplementary Figure S14 Original gel picture of analytical gel filtration fractions of sLCA5-ΔCQS–LC8 and sLCA5-ΔCQS-ΔVQT-LC8 complexes. Lane 1 is the oligomeric fraction of the sLCA5-ΔCQS, and the molar ratio of the LCA5 and the LC8 is 1:1 which indicates a saturated oligomeric complex. Lane 2 is the sample collected from the LCA5 peak fraction with a molar ratio of 2:1. Lane 3 is the sample collected from the LC8 peak. The very first lane and lane 4 is the protein ladder (Thermo Scientific, PageRuler Broad range). Lane 5-6 are the samples collected from the LCA5 fraction of the sLCA5-ΔCQS-ΔVQT complex, with the absence of LC8 in the fraction. Lane 7 is the LC8 fraction of the sLCA5-ΔCQS-ΔVQT complex sample.

The dashed line rectangular markers represent the gel sections used in the densitometric analyses of Figure 6b and 6d.

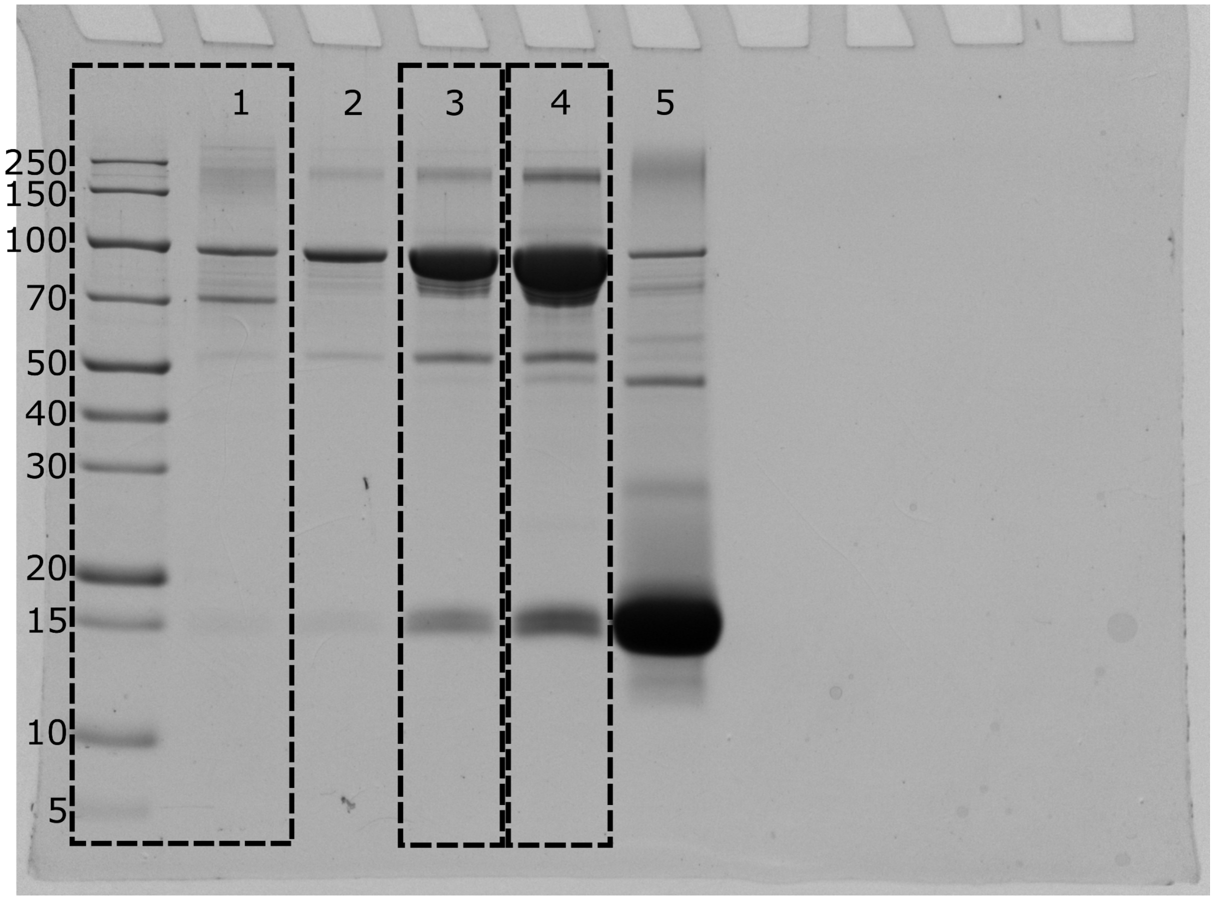

Supplementary Figure S15 Original gel picture of analytical gel filtration fractions of LCA5-ΔVQT-LC8 complex. Lane 1 and 2 are the samples collected from the oligomeric fraction of the LCA5-ΔVQT complex, the fraction contains only a trace amount of LC8 and the estimated molar ratio was less than 2:1. Lane 3 is the sample collected from the first part of the LCA5 peak with a molar ratio of 2:1 which correspond with the molar ratio of a tetrameric non-saturated complex. Lane 4 is the sample collected from the later part of the LCA5 peak, and the molar ratio was 1:1 which corresponds with the molar ratio of a saturated tetrameric complex. Lane 5 is the sample collected from the LC8 fraction of the LCA5-ΔVQT complex. The very first lane is the protein ladder (Thermo Scientific, PageRuler Broad range). The dashed line rectangular markers represent the gel sections used in the densitometric analyses of Figure 6c.

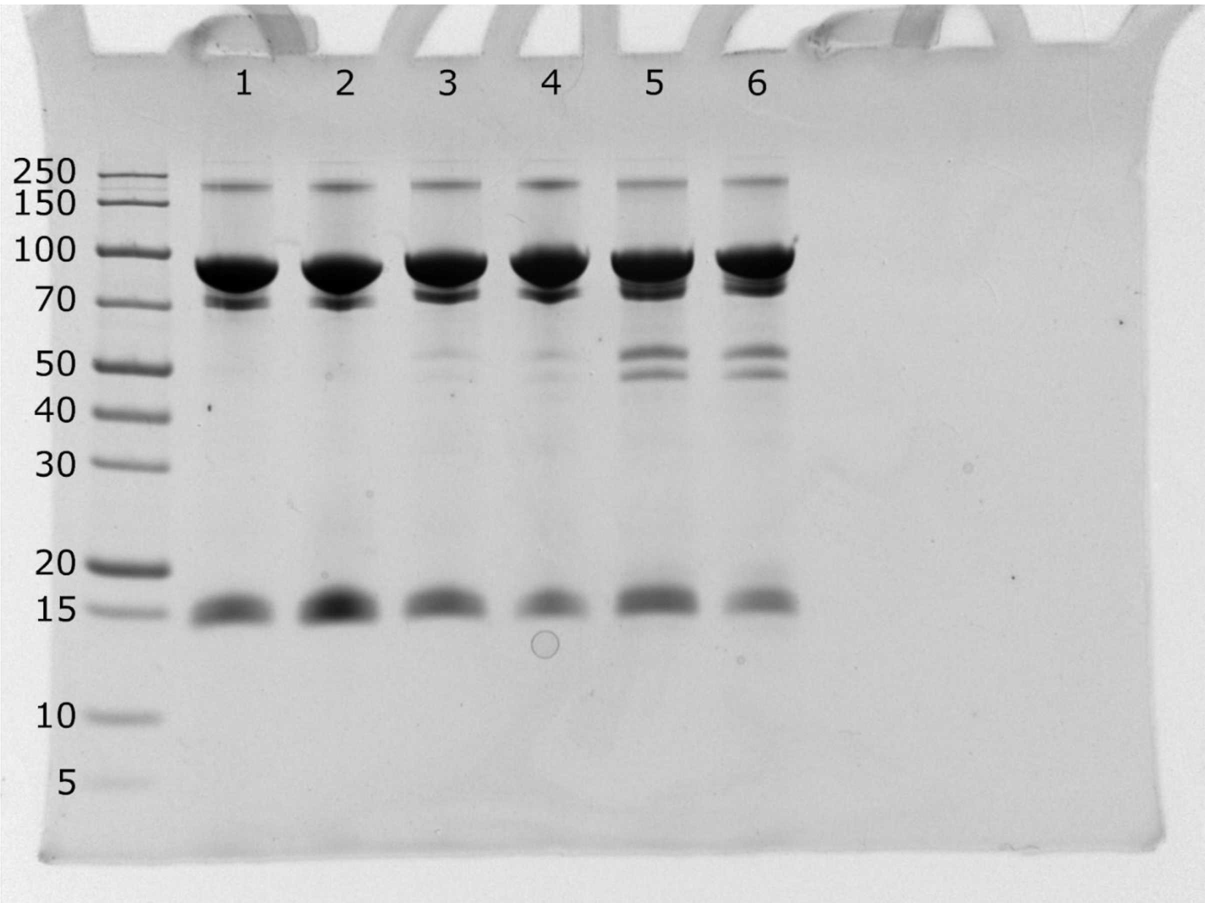

Supplementary Figure S16 Original gel picture of the calibration for densitometry of analytical gel filtration fractions of LCA5-LC8 complexes The first unmarked lane is the protein ladder (Thermo Scientific PageRuler Broad range). Lanes 1-2 are the sLCA5-WT with LC8 in the molar ratio of 1:1 and 1:2. Lane 3-4 are the sLCA5-ΔCQS with LC8 at the molar ratio of 1:1 and 2:1. Lane 5-6 are the sLCA5-ΔVQT with LC8 at the molar ratio of 1:1 and 2:1.

References

1. Oates, M. E. *et al.* D^2P^2: database of disordered protein predictions. *Nucleic Acids Res.* **41,** D508–16 (2013).
2. Erdős, G., Pajkos, M. & Dosztányi, Z. IUPred3: prediction of protein disorder enhanced with unambiguous experimental annotation and visualization of evolutionary conservation. *Nucleic Acids Res.* **49,** W297–W303 (2021).
3. Piovesan, D. *et al.* MobiDB: intrinsically disordered proteins in 2021. *Nucleic Acids Res.* **49,** D361–D367 (2021).
4. Schmidt, H., Zalyte, R., Urnavicius, L. & Carter, A. P. Structure of human cytoplasmic dynein-2 primed for its power stroke. *Nature* **518,** 435–438 (2015).
5. Pettersen, E. F. *et al.* UCSF Chimera--a visualization system for exploratory research and analysis. *J. Comput. Chem.* **25,** 1605–1612 (2004).
6. Kidane, A. I. *et al.* Structural features of LC8-induced self-association of swallow. *Biochemistry* **52,** 6011–6020 (2013).
7. Nyarko, A. & Barbar, E. Light chain-dependent self-association of dynein intermediate chain. *J. Biol. Chem.* **286,** 1556–1566 (2011).
8. Toropova, K. *et al.* Structure of the dynein-2 complex and its assembly with intraflagellar transport trains. *Nat. Struct. Mol. Biol.* **26,** 823–829 (2019).
9. Slevin, L. K., Romes, E. M., Dandulakis, M. G. & Slep, K. C. The mechanism of dynein light chain LC8-mediated oligomerization of the Ana2 centriole duplication factor. *J. Biol. Chem.* **289,** 20727–20739 (2014).
10. Clark, S. *et al.* Multivalency regulates activity in an intrinsically disordered transcription factor. *Elife* **7,** (2018).
